# Safety and effectiveness of adalimumab in patients with rheumatoid arthritis over 5 years of therapy in a phase 3b and subsequent postmarketing observational study

**DOI:** 10.1186/ar4452

**Published:** 2014-01-27

**Authors:** Gerd R Burmester, Marco Matucci-Cerinic, Xavier Mariette, Francisco Navarro-Blasco, Sonja Kary, Kristina Unnebrink, Hartmut Kupper

**Affiliations:** 1 Department of Rheumatology and Clinical Immunology, Charité – University Medicine, Charitéplatz 1, 10117 Berlin, Germany; 2 Azienda Ospedaliera Careggi, Largo Giovanni Alessandro Brambilla, 3, Firenze, Italy; 3 Université Paris-Sud, AP-HP, Hôpital Bicêtre, INSERM U1012, 63, rue Gabriel Péri, Le Kremlin Bicêtre 94276, France; 4 Hospital General, Universitario de Elche, Cami de L'Almassera, 11, 03203 Elche, Alicante, Spain; 5 AbbVie Deutschland GmbH & Co KG, Knollstraße 50, 67061 Ludwigshafen, Germany

## Abstract

**Introduction:**

Patients with active rheumatoid arthritis who had failed at least one disease-modifying anti-rheumatic drug (DMARD) were treated with adalimumab (ADA) in the ReAct study with the option to continue treatment for 5 years in ReAlise. The purpose of this study was to evaluate the long-term safety and effectiveness of ADA as prescribed from the first injection in ReAct to the last observation in ReAlise.

**Methods:**

Patients received ADA alone or in combination with DMARDs according to usual clinical care practices. Adverse events (AEs) were tabulated by five time windows after the first ADA injection. Effectiveness measures included achievement of low disease activity (LDA), defined as Simplified Disease Activity Index (SDAI) ≤11, or remission, (REM), defined as SDAI ≤3.3.

**Results:**

Of the 6,610 ReAct patients, 3,435 (52%) continued in ReAlise. At baseline in ReAct, mean age was 54 years, mean DAS28 was 6.0 and mean HAQ DI was 1.64. The mean treatment duration was 1,016 days, representing 18,272 patient-years (PYs) of ADA exposure. Overall incidence rates of serious AEs and serious infections were 13.8 and 2.8 events (E)/100 PYs, respectively. Serious AEs occurred most frequently in the first 6 months and deceased thereafter. Standardised mortality ratio was 0.71 (95% CI 0.57 to 0.87) and standardised incidence ratio for malignancies was 0.64 (95% CI 0.53 to 0.76). LDA was achieved by 50% and REM by 21% of patients at last observation.

**Conclusions:**

Results of this large observational study of ADA in routine clinical practice were consistent with controlled trials, with no new safety concerns during a follow-up of more than 5 years. Effectiveness of ADA was maintained during long-term observation.

**Trial registration:**

NCT00448383, NCT00234884

## Introduction

Patients with rheumatoid arthritis (RA) may not respond to treatment with disease-modifying anti-rheumatic drugs (DMARDs) alone [1-4]. In patients who have failed DMARD therapy for RA, clinical studies have demonstrated the effectiveness of drugs directed against tumour necrosis factor (TNF) as monotherapy or when used in combination with DMARDs [2,5-10]. Adalimumab (ADA) is a fully human anti-TNF monoclonal antibody for the treatment of moderate to severe RA. Initial clinical trials of ADA in patients with RA demonstrated a good safety profile, with improvements in disease signs and symptoms and functional ability, achievement of clinical remission and inhibition of radiographic disease progression [2,3,7].

The Research in Active Rheumatoid Arthritis (ReAct) phase 3b study was initiated in 2002 to assess the safety and effectiveness of ADA in RA patients who had failed treatment with at least one traditional DMARD [11,12]. ADA was well tolerated and effective, alone or with DMARDs, in 6,610 patients with active RA over a mean treatment duration of 233 days [11,12]. To evaluate the long-term safety and effectiveness of ADA in clinical practice settings over 5 years in patients who completed ReAct, the REgistry of HUMIRA™ in RA: a Long-Term Investigation of Safety and Efficacy (ReAlise) observational follow-up study was conducted (NCT00234884). The primary objectives of this analysis include examination of adverse events (AEs) and the temporal pattern of their occurrence and maintenance of response through 5 years of ADA treatment (i.e., from the first injection received in ReAct through the last observation in ReAlise).

## Methods

### Study design

ReAct was a 12-week, open-label multicentre study with an optional extension phase until ADA became commercially available. Methodology and results have been published [11]. Briefly, ADA was administered to 6,610 patients with active RA (defined as 28-joint Disease Activity Score (DAS28) based on erythrocyte sedimentation rate (ESR) ≥3.2 and an unsatisfactory response to at least one synthetic DMARD). Patients also could have received prior TNF antagonist therapy with infliximab and/or etanercept if treatment was stopped 2 months before inclusion in ReAct. Patients were allowed to continue treatment with DMARDs, corticosteroids and non-steroidal anti-inflammatory drugs (NSAIDs). Subsequently, patients could enter ReAlise, a multicentre (10 European countries and Australia), 5-year, uncontrolled observational study of ADA in patients with long-standing, severe RA. Patients were treated in accordance with physicians’ usual clinical care practices and local marketing authorisation requirements for commercially available ADA. ReAlise was conducted as a commitment to the European Medicines Agency (EMEA) and in accordance with the Declaration of Helsinki and applicable local regulations; each site’s institutional review board or independent ethics committee approved the protocol (Additional file 1 and Additional file 2 for ReAlise and ReAct, respectively), and all patients provided written informed consent.

### Patients

Patients aged ≥18 years were eligible for ReAlise if they were in good health (per physician’s discretion) with a recent stable medical history, receiving ongoing ADA treatment, completed ≥3 months of the ReAct study, and were prescribed ADA. Patients could enroll in ReAlise within 12 months of completing ReAct. Patients continued taking concomitant medications, including DMARDs and corticosteroids, per usual care. Assessments were made at weeks 2, 6 and 12, and every 8 weeks thereafter in ReAct, every 3 months during the patients’ first year in ReAlise and every 6 months thereafter until the last observation in ReAlise.

### Assessments

This analysis includes clinical assessments of safety and effectiveness made at 0.5, 1, 3 and 5 years and the last visit after the first ADA injection in ReAct. AEs were collected throughout ReAct and ReAlise and for 3 months after the last visit in ReAlise. There was no systematic collection of AEs during the interval between the last assessment in ReAct and enrolment in ReAlise. AEs were tabulated using five time windows (≤0.5, 0.5 to 1, >1 to 3, >3 to 5 and >5 years) beginning after the first injection of ADA in ReAct. Standardised mortality rates (SMRs) and standardised incidence rates (SIRs) for malignancies were calculated for all patients, for all completed treatment periods.

Clinical effectiveness was assessed by the following measures: American College of Rheumatology improvements of 20% (ACR20), 50% (ACR50) and 70% (ACR70) [13]; European League Against Rheumatism (EULAR) categorical responses of moderate and good [14]; DAS28 (ESR) including individual components: swollen and tender joint counts (SJC, TJC), patient global assessment (PtGA), and ESR; low disease activity (LDA) and remission (REM) were assessed using the Simplified Disease Activity Index (SDAI) scores of ≤11 or ≤3.3, respectively [15]. Physical functioning was measured using the Health Assessment Questionnaire Disability Index (HAQ DI). Data were analysed for mean HAQ DI scores, the percentage of patients with minimal important difference ≥0.22 and the percentage of patients with normal function, defined as HAQ DI ≤0.5.

### Statistical analysis

Data were integrated from ReAct and ReAlise to evaluate long-term ADA treatment. Safety and effectiveness analyses were performed on the intention-to-treat (ITT) population and included all patients who had at least one ADA injection in ReAct. AEs were reported as events (E) and E per 100 patient-years (PYs) for all treated patients. For SMRs, study results were compared against age- and sex-matched data from the World Health Organization for each country in which the study was conducted [16]. SIRs for malignancies were determined by comparing study results with the National Cancer Institute (NCI) Surveillance, Epidemiology and End Results (SEER) database for all malignancies and lymphoma [17].

To examine the effect of different therapies on the incidence of AEs, subgroup analyses were conducted for patients receiving ADA monotherapy (defined as no concomitant DMARD irrespective of combination with corticosteroids or NSAIDs) versus combination therapy of ADA with at least one DMARD. Patients also were stratified by use of corticosteroids and prior use of TNF antagonists (infliximab or etanercept) versus those who had not used these medications.

Mean (standard deviation (SD)) values were reported for clinical effectiveness measures with continuous data (for example, DAS28) using observed values. Categorical data (for example, ACR, EULAR responses) were summarised with absolute and relative frequencies using observed values.

## Results

### Patients

**Figure 1:**
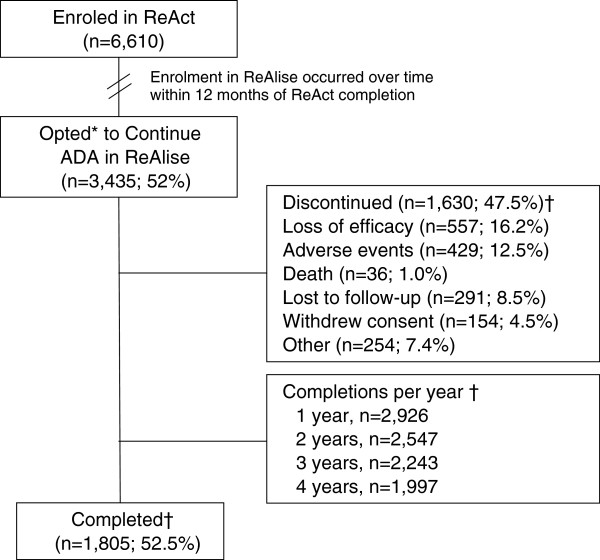
**Patient disposition.** *ReAct was followed by ReAlise when ADA was commercially licensed, giving patients the opportunity to be further observed for safety and effectiveness over 5 years or more. †Discontinuations and completions are listed for the entire duration of the ReAlise study. ADA, adalimumab; ReAct, the Research in Active Rheumatoid Arthritis phase 3b study; ReAlise, the Registry of HUMIRA™ in RA: a Long-Term Investigation of Safety and Efficacy observational follow-up study.

Of the 6,610 patients in ReAct, 3,435 (52%) opted to continue ADA, as prescribed by their physician, in ReAlise (Figure 1). Of the 3,435 patients who continued in ReAlise, 1,805 (52.5%) completed the long-term study, receiving 5 years or more of ADA. Primary reasons for discontinuation in ReAlise were loss of efficacy (n = 557, 16.2%) or AEs (n = 429, 12.5%). Withdrawals were generally evenly distributed across time intervals, without obvious clusters of withdrawals occurring for specific reasons. AEs led to study withdrawal in approximately 2.5% of patients during the first 6 months and from 6 to 12 months and decreased to 1 to 22% during subsequent 6-month intervals through 60 months. Lack of efficacy was cited as a reason for study withdrawal in 2 to 3% of patients for all 6-month intervals.

Patient demographics and baseline clinical characteristics are shown in Table 1. Moderate to severe disease activity and functional impairment are demonstrated by mean DAS28 (ESR) of 6.0 and mean HAQ DI score of 1.64. Before entry into ReAct, 97% of patients had taken one or more DMARD. The percentages of patients who had received methotrexate, leflunomide, antimalarials (chloroquine or hydroxychloroquine), and sulfasalazine were 89%, 42%, 42%, and 39%, respectively [11]. Etanercept and/or infliximab were previously prescribed for 899 (13.6%) patients [12]. Reasons for discontinuing TNF antagonists before ReAct were loss of response (38% (327/870)), no primary response (22% (195/870)) and intolerance (22% (190/870)) [12]. At baseline of ReAct, 74% (4,879/6,610) of patients were taking at least one DMARD and 71% (4,708/6,610) were taking corticosteroids [11]. At first visit in ReAlise, most patients (62%) were taking only one DMARD, and the most common agents were methotrexate (58%), leflunomide (13%), antimalarials (8%) and sulfasalazine (6%). ADA was administered as monotherapy for 26% of patients. Aside from DMARDs, corticosteroids were the most frequently administered concomitant medications. Of 3,175 patients who completed ReAct but did not continue in ReAlise, demographics and baseline clinical characteristics were similar to the population that continued in ReAlise (data not shown).

**Table 1:** Baseline demographics and disease activity

	**At baseline in ReAct**		**At baseline in ReAlise**	
**Parameter**	**n**	**Value**	**n**	**Value**
Age, y, mean (SD)	6,610	53.7 (13.0)	3,435	54.5 (12.5)
Female, n (%)	6,610	5,332 (80.7)	3,435	2,724 (79.3)
Duration of RA, y, mean (SD)	6,572	10.8 (8.6)	3,423	12.0 (8.6)
Rheumatoid factor positive, n (%)	6,610	4,811 (72.8)	3,429	2,548 (74.2)*
DMARD use at baseline, n (%)	6,610	4,879 (73.8)	3,435	2,636 (76.7)*
SJC (28), mean (SD)	6,607	10.4 (5.8)	3,435	2.2 (3.4)
TJC (28), mean (SD)	6,607	13.5 (7.0)	3,435	3.2 (4.7)
PtGA, mm VAS, mean (SD)	6,568	61.9 (21.4)	3,256	26.2 (23.3)
DAS28 (ESR), mean (SD)	6,554	6.0 (1.1)	2,729	3.5 (1.3)
HAQ DI, mean (SD)	6,560	1.64 (0.68)	3,235	0.89 (0.74)
CRP, mg/L, mean (SD)	6,535	25.7 (30.8)	2,962	9.5 (15.7)

*At time of entry into ReAct study. CRP, C-reactive protein; DAS28, 28-joint Disease Activity Score; DMARD, disease-modifying anti-rheumatic drug; ESR, erythrocyte sedimentation rate; HAQ DI, Health Assessment Questionnaire Disability Index; PtGA, patient global assessment; RA, rheumatoid arthritis; SD, standard deviation; SJC, swollen joint count; TJC, tender joint count; VAS, visual analogue scale.

From the first dose of ADA in ReAct, the mean (SD) and median treatment durations were 1,016 (895) and 546 days, respectively (range, 14 to 2,681 days) or 2.9 (2.5) and 1.5 years, representing 18,272 PYs of exposure. The mean (SD) and median intervals between completion of ReAct and enrolment in ReAlise were 5.5 (3.4) and 4.8 months, respectively.

### Safety

Overall, 81.8% of patients experienced one or more treatment-emergent AEs at any time from the first injection in ReAct to the end of ReAlise, with an overall AE incidence rate of 137.7 E/100 PYs. The overall incidence rate of serious adverse events (SAEs) was 13.8 E/100 PYs (Table 2), with SAEs reported more frequently during the first 6 months of treatment and decreasing thereafter. The overall incidence rate of serious infections was 2.8 E/100 PYs, and this rate also decreased over time. Regarding AEs of special interest in RA, rates of tuberculosis (TB), malignancies, lymphoma, non-melanoma skin cancer (NMSC), serious congestive heart failure, cerebrovascular events and serious hepatic events decreased over the period from 1 year to more than 5 years (Table 2). Of the 35 total cases of TB, 16 (45.7%) were extra-pulmonary.

**Table 2:** Overview of serious adverse events and adverse events of interest (E (E/100 PYs)) during more than 5 years of ADA treatment

		**Time windows after the first injection of ADA in ReAct**				
		**≤0.5 Y**	**>0.5 to 1 Y**	**>1 to 3 Y**	**>3 to 5 Y**	**>5 Y**
		**N = 6,610**	**N = 5,922**	**N = 4,283**	**N = 2,623**	**N = 2,000**
**Adverse event (AE)**	**Overall N = 6,610 (18,272 PYs)**	**(3,059 PYs)**	**(2,256 PYs)**	**(6,149 PYs)**	**(4,549 PYs)**	**(2,260 PYs)**
Serious AEs*	2,529 (13.8)	838 (27.4)	419 (18.6)	661 (10.7)	417 (9.2)	194 (8.6)
Fatal AEs	102 (0.6)	29 (0.9)	19 (0.8)	27 (0.4)	17 (0.4)	10 (0.4)
Serious infections*	518 (2.8)	162 (5.3)	83 (3.7)	154 (2.5)	81 (1.8)	38 (1.7)
TB^†^	35 (0.2)	11 (0.4)	11 (0.5)	8 (0.1)	4 (0.1)	1 (<0.1)
Sepsis	35 (0.2)	13 (0.4)	4 (0.2)	7 (0.1)	7 (0.2)	4 (0.2)
Malignancies^‡^	121 (0.7)	19 (0.6)	16 (0.7)	45 (0.7)	25 (0.5)	16 (0.7)
Lymphoma	15 (0.1)	1 (<0.1)	0	9 (0.1)	4 (0.1)	1 (<0.1)
NMSC	43 (0.2)	8 (0.3)	2 (0.1)	17 (0.3)	11 (0.2)	5 (0.2)
Serious CHF*	47 (0.3)	15 (0.5)	6 (0.3)	12 (0.2)	13 (0.3)	1 (<0.1)
Cerebrovascular AEs^§^	56 (0.3)	13 (0.4)	5 (0.2)	16 (0.3)	15 (0.3)	7 (0.3)
Serious hepatic events*	58 (0.3)	10 (0.3)	13 (0.6)	16 (0.3)	13 (0.3)	6 (0.3)

*Serious adverse events include those that met any of the following criteria: death, life-threatening (that is, would have resulted in immediate fatality without medical intervention), hospitalisation, prolongation of hospitalisation, and persistent or significant disability, or any important medical event requiring medical or surgical intervention to prevent serious outcome. ^†^Including two patients with a positive test for latent TB during ADA therapy. ^‡^Excluding, lymphomas and NMSC. These 121 events occurred in 114 subjects. ^§^Such as stroke and transient ischemic attack. ADA, adalimumab; CHF, congestive heart failure; E, events; NMSC, non-melanoma skin cancer; PYs, patient-years; TB, tuberculosis.

SIR was 0.64 (95% confidence interval (CI) 0.53 to 0.76) for all malignancies (118 observed^a^/185.48 expected), excluding NMSC, and 1.99 (95% CI 1.11 to 3.28) for lymphomas (15 observed/7.55 expected). SIR for melanoma was 1.29 (95% CI 0.59 to 2.54; 8 observed/6.20 expected). Overall SMR was 0.71 (95% CI 0.57 to 0.87); there were fewer observed deaths during ADA exposure (90) than expected (127). The SMR was 0.60 (95% CI 0.38 to 0.89) for men and 0.76 (95% CI 0.59 to 0.96) for women. For both men and women, fewer deaths were observed (23 and 67, respectively) than were expected (39 and 88, respectively).

### Subgroup safety analyses

The incidences of SAEs were slightly higher among patients who received ADA monotherapy (no DMARDs) and those who received corticosteroids (17.4 and 14.9 E/100 PYs, respectively) than for those who received at least one concomitant DMARD and those who did not receive corticosteroids (12.8 and 11.5 E/100 PYs, respectively). The rates of serious infections were generally similar. For patients who had received prior TNF antagonists, the overall incidence of serious infections was higher (4.2 E/100 PYs) than that for patients who had not received prior TNF antagonists (2.7 E/100 PYs). All other AEs of interest, including malignancies and lymphomas, were reported at similar rates for patients with and without a history of exposure to TNF antagonists at study entry. Similar to the overall population, SAEs and AEs of interest occurred more frequently during the initial 6 months of therapy and decreased thereafter in all subgroups (see Tables S1-S6 in Additional file 3 for data).

### Effectiveness

**Figure 2:**
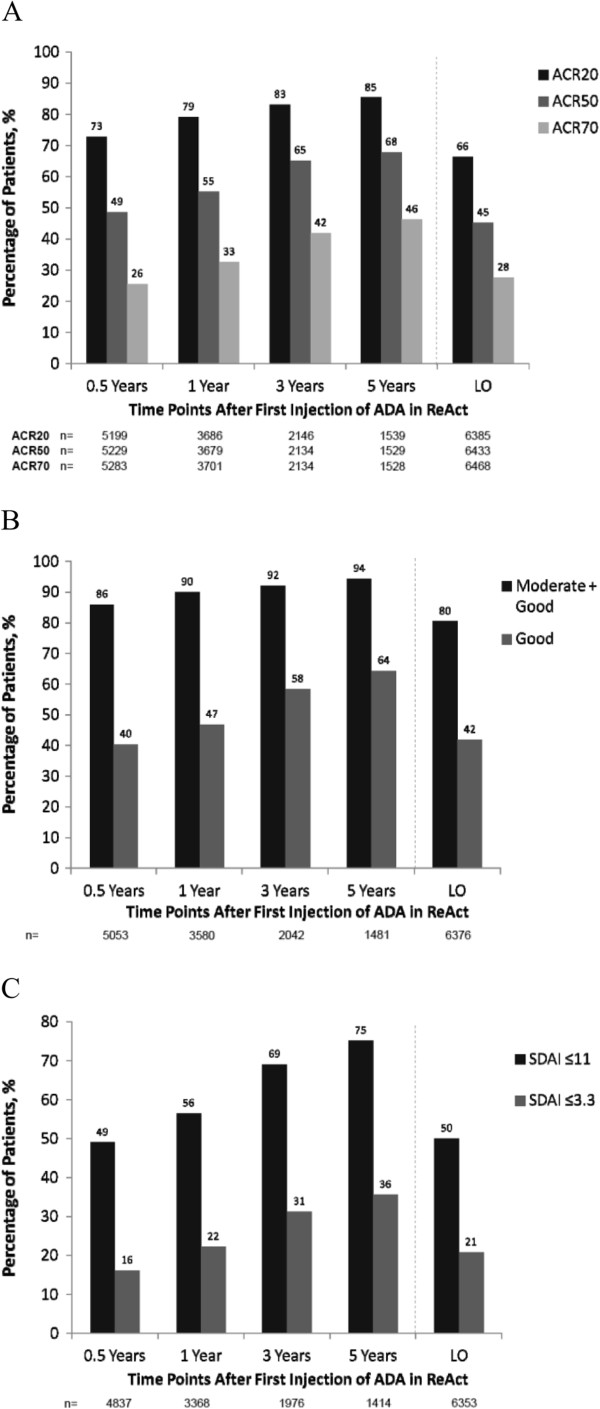
**Percentages of patients with (A) American College of Rheumatology (ACR) 20%, 50%, or 70% improvement; (B) European League Against Rheumatism (EULAR) responses of at least ‘moderate’ and ‘good’; and (C) low disease activity (LDA) and disease remission (REM) defined as Simplified Disease Activity Index (SDAI) ≤11 and SDAI ≤3.3, respectively.** Data are shown as observed values for all evaluable patients at each time point during long-term treatment with adalimumab (ADA). LO, last observation.

Over more than 5 years of ADA treatment, ACR and EULAR categorical responses were maintained (Figure 2A and 2B). At last observation, ACR20, ACR50 and ACR70 responses were achieved by 66%, 45% and 28%, respectively, and moderate and good EULAR responses were achieved by 80% and 42%, respectively. The percentages of patients achieving the stringent ACR/EULAR index-based definitions of LDA and REM based on SDAI were sustained over the course of the study, with 50% achieving LDA at last observation (Figure 2C). Approximately one in five patients achieved REM at last observation, while more than one-third of patients who completed 5 years of ADA achieved REM.

**Figure 3:**
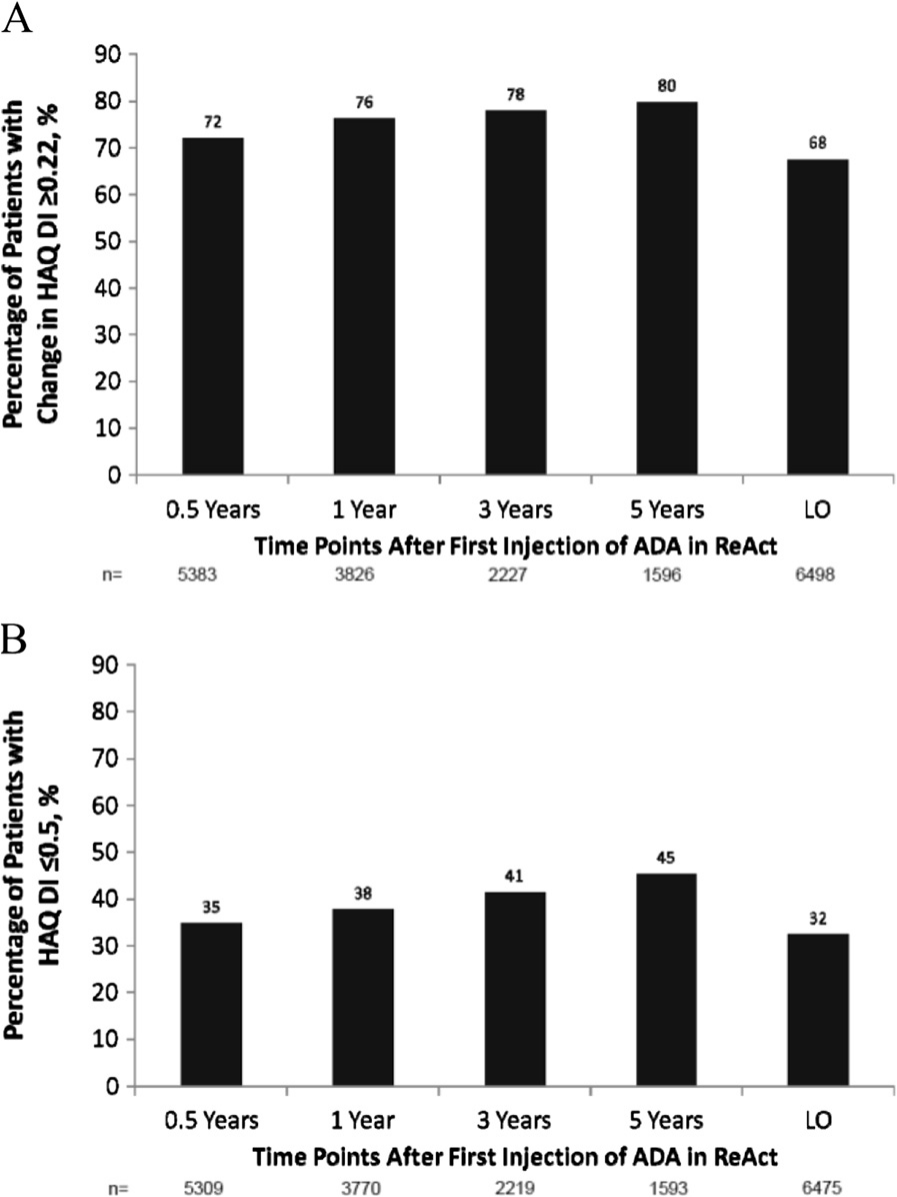
**Percentages of patients with (A) minimal important difference ≥0.22 on the Health Assessment Questionnaire Disability Index (HAQ DI) and (B) normal function defined as HAQ DI ≤0.5 over long-term adalimumab (ADA) treatment.** Observed data are reported. LO, last observation.

**Figure 4:**
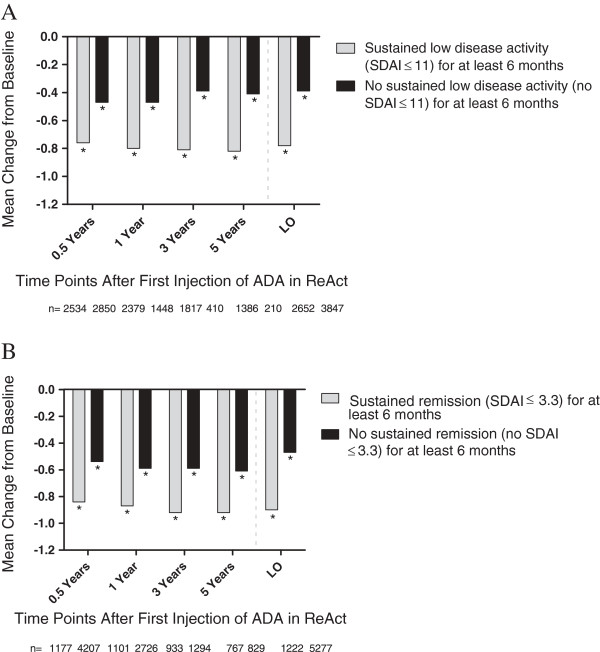
**Mean change of Health Assessment Questionnaire Disability Index (HAQ DI) from baseline in patients (A) with sustained low disease activity (LDA) defined as Simplified Disease Activity Index (SDAI) ≤ 11 for at least 6 months and without sustained LDA and (B) with sustained remission (REM) defined as SDAI ≤ 3.3 for at least 6 months and without sustained REM.** Data are shown as observed values for all evaluable patients at each time point during long-term treatment with adalimumab (ADA). *Denotes significance from baseline, *P* ≤0.001. LO, last observation.

Mean DAS28 (ESR) scores and individual component values showed consistent improvements throughout treatment with ADA (Table 3). Reductions in mean HAQ DI score were observed at 0.5 years and remained stable throughout the more than 5-year duration of the study (Table 3). The proportion of patients who achieved clinically relevant reductions in physical disability at last observation was 68% and showed consistency over 5 years, ranging from 72 to 80% (Figure 3A). The proportion of patients with normal function (HAQ DI ≤0.5) was 32% at last observation and ranged from 35 to 45% over 5 years of ADA treatment (Figure 3B). For those patients who had achieved either sustained LDA (SDAI ≤11) or sustained REM (SDAI ≤3.3) for at least 6 months, there was a greater reduction in mean HAQ DI than for those patients without sustained LDA or REM (Figure 4).

**Table 3:** DAS28 (ESR) composite and individual component values, and SDAI and HAQ DI scores at 0.5, 1, 3 and 5 years of ADA exposure and last observation

**Parameter**	**0.5 Y**	**1 Y**	**3 Y**	**5 Y**	**LO**
DAS28 (ESR)					
Mean (SD)	3.6 (1.5)	3.4 (1.4)	3.1 (1.3)	2.9 (1.2)	3.8 (1.6)
Median (IQR)	3.5 (2.0)	3.3 (2.0)	2.9 (1.8)	2.8 (1.5)	3.6 (2.4)
PtGA, mm VAS					
Mean (SD)	30.6 (24.6)	27.8 (23.7)	25.1 (22.9)	23.4 (21.6)	34.5 (27.1)
Median (IQR)	25.0 (37.0)	22.0 (35.0)	19.0 (32.0)	18.0 (30.3)	30.0 (44.0)
TJC (28)					
Mean (SD)	4.1 (5.4)	3.2 (4.6)	2.4 (4.1)	1.9 (3.6)	4.6 (6.3)
Median (IQR)	2.0 (6.0)	2.0 (4.0)	1.0 (3.0)	0.0 (2.0)	2.0 (6.0)
SJC (28)					
Mean (SD)	3.0 (4.1)	2.3 (3.5)	1.4 (2.8)	1.1 (2.5)	3.1 (4.6)
Median (IQR)	2.0 (4.0)	1.0 (3.0)	0.0 (2.0)	0.0 (1.0)	1.0 (4.5)
ESR, mm/h					
Mean (SD)	24.1 (20.0)	23.1 (19.3)	21.7 (17.5)	21.3 (17.3)	26.9 (22.2)
Median (IQR)	18.0 (22.0)	18.0 (20.0)	17.0 (21.0)	17.0 (19.0)	20.0 (27.0)
SDAI					
Mean (SD)	13.7 (12.1)	11.4 (10.6)	8.8 (9.0)	7.6 (8.2)	14.7 (14.2)
Median (IQR)	10.3 (13.5)	8.5 (11.9)	6.1 (9.3)	5.2 (8.0)	10.1 (16.8)
HAQ DI score					
Mean (SD)	1.01 (0.78)	0.94 (0.76)	0.85 (0.73)	0.80 (0.72)	1.09 (0.82)
Median (IQR)	1.00 (1.3)	0.88 (1.3)	0.75 (1.1)	0.63 (1.1)	1.00 (1.4)

ADA, adalimumab; DAS28, 28-joint Disease Activity Score; ESR, erythrocyte sedimentation rate; HAQ DI, Health Assessment Questionnaire Disability Index; IQR, interquartile range; LO, last observation; PtGA, patient global assessment; SD, standard deviation; SDAI, Simplified Disease Activity Index; SJC, swollen joint count; TJC, tender joint count; VAS, visual analogue scale.

For patients who had received prior TNF antagonists (infliximab or etanercept), both the ACR and EULAR responses were slightly lower than those patients who had not received prior TNF antagonists (Figure S1 in Additional file 4 and Figure S2 in Additional file 5). At last observation the ACR20, ACR50, and ACR70 responses were achieved by 68%, 47%, and 29% in those without prior TNF antagonists and 55%, 32%, and 17% in the patients who had received prior TNF antagonists. The percentages of patients achieving the stringent ACR/EULAR index-based definitions of LDA and REM based on SDAI were also slightly less for the patients who had previous TNF antagonists (Figure S3 in Additional file 6). Additionally, the proportion of patients with normal function (HAQ DI ≤0.5) was 34% at last observation for those without prior TNF antagonists and 19% in the patients who had received prior TNF antagonists.

## Discussion

The combined data from ReAct and ReAlise include more than 6,600 RA patients and provide one of the largest well-monitored evaluations of the long-term safety and effectiveness of a single TNF antagonist in routine clinical practice settings. Other reports involving thousands of RA patients come from country registries that record and compare use of multiple TNF antagonists [18-22]. The observational study design allows for inclusion of patients receiving routine care for RA and is not restricted to patients who meet the stricter entry criteria of randomised controlled trials, including fewer comorbidities. Patients enrolled in ReAct and ReAlise reflect typical RA patients treated with TNF antagonists, who have long-standing RA with moderate to severe baseline disease activity despite treatment with DMARDs. Some patients also had previously received infliximab and/or etanercept.

ADA was well tolerated, and no new or unexpected safety concerns were identified during more than 5 years of therapy, representing nearly 20,000 PYs. The pattern and rates of AEs were comparable with those observed in double-blind studies of ADA with or without methotrexate [2,3,7]. SAEs, including serious infections and TB, were reported more frequently during the first year, after which the incidences decreased and remained stable. This finding is expected because of positive selection bias of the patients who tolerate the drug and less frequent visits in ReAlise, and generally aligns with data from ADA clinical trials and registries [4,7,18,23-25]. A recent review of safety data from ADA clinical studies across indications found a stable rate of serious infections throughout ADA exposure, which may be attributed to differences in populations and methodology [26]. Beyond the positive selection bias of the patients who tolerate the drug, the other possible explanations for the decrease in infection risk with ongoing TNF antagonist therapy may include decreased use of corticosteroids and better control of the RA disease process that reduces disease-associated alterations in natural immunity [18,19]. The overall incidence rates of SAEs and serious infections with ADA (13.8 and 2.8 E/100 PYs) were found to be within the range of those previously reported in long-term clinical trials of other TNF antagonists and biological DMARDs. Rates of serious infections for infliximab and etanercept after a median follow-up of 3.9 years in the British registry were 4.6 and 3.8 E/100 PYs, respectively [18]. Five-year data on abatacept and tocilizumab report serious infection rates of 3.0 and 5.7 E/100 PYs, respectively [27,28]. SAEs of interest other than serious infections occurred at a low frequency and decreased over time with ADA. Similarly, the risk of TB decreased over time, with an incidence rate <0.1% in the patients treated more than 5 years. Still, five cases occurred in patients treated for more than 3 years, underscoring the need for continued attention to the risk of infection. There was no overall increased risk of malignancies; however, use of a US database (NCI) as a comparator for SIRs in this largely European population may limit interpretation of results. The observed risk of lymphomas was consistent with that for RA patients. The risk of lymphoma, both Hodgkin’s and non-Hodgkin’s, is increased by approximately two-fold in patients with RA, and this increase has been associated with the chronic inflammation of RA [23,29,30]. Use of disease-modifying treatment that controls chronic inflammation may reduce the elevated risk associated with uncontrolled RA. Recent long-term studies have failed to identify an effect of TNF antagonists on the incidence of cancer, including lymphoma (with exception of NMSC), and also determined that the relative risk of cancer did not increase with time since first starting a TNF antagonist or with cumulative duration of TNF antagonist therapy [31-33]. Lastly, mortality rates were lower in patients receiving ADA therapy in ReAct and ReAlise than observed in a matched population without RA, and there was no increase in the overall mortality rate over the course of the studies. The decreased rates of cancer and mortality must be interpreted with caution because patients with previous cancers may have been excluded from receiving a TNF antagonist.

Patients were allowed to continue concomitant DMARD therapy and/or corticosteroids with ADA in ReAct and ReAlise. Generally, the incidences of SAEs were similar among subgroups based on concomitant therapy, with a slightly higher rate among patients who received ADA monotherapy (no DMARDs) and those who received corticosteroids (17.4 and 14.9 E/100 PYs, respectively). Patients who had received etanercept and/or infliximab before enrolling in ReAct showed a higher rate of SAEs and infections than patients without prior history of TNF antagonist therapy, but no differences in the rates of malignancies or lymphoma were observed. Statistical analyses of these subgroups were not performed in this observational study; however, no statistical differences have been identified in controlled clinical studies that evaluated AEs in subgroups based on prior or concomitant treatment [2,3,12].

The effectiveness of ADA in reducing disease activity and inflammation was maintained over more than 5 years of treatment, as evidenced by the percentages of patients achieving ACR20, 50 and 70 responses and at least moderate EULAR responses. The initial 12-week improvements observed in ReAct included ACR20 in 69% and moderate and good EULAR responses in 83% and 33%, respectively [11]. For patients who continued in ReAlise, these response rates were 66%, 80% and 42%, respectively, at last observation. LDA and REM rates also were maintained at last observation, with half of the patients achieving LDA, and one in five achieving REM. Clinically relevant functional improvement was reported for approximately two-thirds of patients, and one-third of patients reported normal function at last observation.

Approximately 70% of patients who received prior TNF antagonists discontinued that therapy because of loss or lack of response before enrolling in ReAct/ReAlise. At week 12 of ReAct, substantial clinical benefit was achieved with ADA in patients previously treated with infliximab and/or etanercept [12], and response rates were maintained through 3 years of ADA [34]. In agreement with our data, clinical studies that have evaluated the effectiveness of switching TNF antagonists have generally found that treatment with a second TNF antagonist can offer comparable or slightly lower response rates than those observed in TNF antagonist-naive patients [20,35,36]. However, as the number of TNF antagonists increase to three or more, response rates decrease. In a study of more than 2,000 RA patients, discontinuation rates were similar for infliximab, etanercept and ADA, and factors predictive of discontinuation were increased disease severity and comorbidities [36]. Delaying administration of TNF antagonists in patients who fail to achieve an adequate response with methotrexate has been associated with poorer clinical, functional and radiographic outcomes [4,37]. The findings from ReAct/ReAlise indicate that switching from failed therapy with a traditional DMARD or TNF antagonist to ADA (with or without concomitant DMARD) is a reasonable strategy.

Limitations of long-term observational studies include inherent bias. Patients who experience effective or tolerable therapy are likely to continue treatment. In ReAct/ReAlise, the most common reasons for patients discontinuing the study were loss of efficacy and AEs. To address potential bias, assessment of effectiveness over 5 years included results for all efficacy parameters at last observation. Results at last observation were consistent with results at the end of 12-week treatment in ReAct [11].

## Conclusions

In a clinical practice setting, ADA was well tolerated, and no new safety concerns were identified during nearly 20,000 PYs of exposure. The incidence of SAEs and serious infections decreased with ongoing ADA treatment over 5 years. The incidences of deaths and malignancies were lower than expected for the general population, and the incidence of lymphoma was within expected rates for patients with RA. Clinical response to ADA, as measured by reduced disease activity and functional improvements, was maintained through more than 5 years of observation in patients with long-standing, severe RA. Safety and effectiveness observed in more than 6,600 RA patients over 5 years were consistent with results of randomised clinical trials of ADA [2,3,7].

## Endnotes

^a^From a total of 129 cases (114 cases of malignancy (excluding lymphomas, and NMSC) plus 15 cases of lymphoma), 11 were excluded (9 cases of carcinoma *in situ* and 2 of metastases for which the primary cancer was already counted), in keeping with the SEER database criteria for comparison.

## Abbreviations

ACR: American College of Rheumatology; ADA: adalimumab; AE: adverse event; CI: confidence interval; DAS28: 28-joint Disease Activity Score based on ESR; DMARD: disease-modifying anti-rheumatic drug; E: event; EMEA: European Medicines Agency; ESR: erythrocyte sedimentation rate; EULAR: European League Against Rheumatism; HAQ DI: Health Assessment Questionnaire Disability Index; ITT: intention-to-treat; LDA: low disease activity; NCI: National Cancer Institute; NMSC: non-melanoma skin cancer; NSAID: non-steroidal anti-inflammatory drug; PtGA: patient global assessment; PYs: patient-years; RA: rheumatoid arthritis; REM: remission; SAE: serious adverse event; SD: standard deviation; SDAI: Simplified Disease Activity Index; SEER: Surveillance, Epidemiology and End Results; SIR: standardised incidence ratio; SJC: swollen joint count; SMR: standardised mortality ratio; TB: tuberculosis; TJC: tender joint count; TNF: tumour necrosis factor.

## Competing interests

The ReAct and ReAlise studies were funded by AbbVie Inc, which participated in designing and conducting the study; collection, analyses, and interpretation of data; and writing, reviewing, and approval of the publication. GRB received consulting fees and payment for speakers bureaus and board membership from AbbVie, Bristol-Myers Squibb (BMS), MSD, Pfizer, Roche, and UCB. GRB’s institution has received research grants from AbbVie, BMS, MSD, Pfizer, Roche, and UCB. MM-C declares that he has no competing interests. XM received research grants from Pfizer and Roche and consulting fees from BMS, GSK, LFB, Pfizer, Roche and UCB. FN-B received research grants from Roche. SK is a former contract employee for AbbVie. KU is an employee of AbbVie and may hold AbbVie stock. HK is an employee of AbbVie, may hold AbbVie stock, and is named an inventor on the following patent applications assigned to AbbVie Biotechnology Ltd: WO2011/097301 published 8/11/2011, WO2007/120656 published 10/25/2007, WO2007/120626 published 10/25/2007, US20120171123A published 1/5/2012.

## Authors’ contributions

GRB made substantial contributions to conception and design, acquisition of data, analysis and interpretation of data; was involved in drafting the manuscript and revising the manuscript critically for important intellectual content; and has given final approval of the version to be published. MM-C made substantial contributions to acquisition of data and analysis and interpretation of data; was involved in revising the manuscript critically for important intellectual content; and has given final approval of the version to be published. XM made substantial contributions to acquisition of data and analysis and interpretation of data; was involved in revising the manuscript critically for important intellectual content; and has given final approval of the version to be published. FN-B made substantial contributions to acquisition of data; was involved in drafting the manuscript and revising the manuscript critically for important intellectual content; and has given final approval of the version to be published. SK made substantial contributions to conception and design, analysis and interpretation of data; was involved in drafting the manuscript and revising the manuscript critically for important intellectual content; and has given final approval of the version to be published. KU made substantial contributions to conception and design, acquisition of data, analysis and interpretation of data; was involved in drafting the manuscript and revising the manuscript critically for important intellectual content; and has given final approval of the version to be published. HK made substantial contributions to conception and design, and analysis and interpretation of data; was involved in drafting the manuscript and revising the manuscript critically for important intellectual content; and has given final approval of the version to be published. All authors read and approved the final manuscript.

## Supplementary Material

Additional file 1 **Name and address of the Independent Ethics Committee/Institutional Review Board (IEC/IRB) from the ReAlise Study (NCT00234884).** Click here for file

Additional file 2 **Name and address of the Independent Ethics Committee/Institutional Review Board (IEC/IRB) from the ReAct Study (NCT00448383).** Click here for file

Additional file 3: Table S1-S6 Overview of serious adverse events and adverse events of interest (E (E/100 PYs)) in patients who recieved no concomitant disease-modifying anti-rheumatic drugs (DMARDs), at least 1 concomitant DMARD, no concomitant corticosteriods, concomitant corticosteriods, no prior infliximab or etanercept, and prior infliximab or etanercept, respectively. E, events; PYs, patient-years. Click here for file

Additional file 4: Figure S1 Percentages of patients with American College of Rheumatology (ACR) **(A)** 20%, **(B)** 50%, or **(C)** 70% improvement for those patients with prior use of TNF antagonists and TNF antagonist-naive patients. Data are shown as observed values for all evaluable patients at each time point during long-term treatment with adalimumab (ADA). LO, last observation. Click here for file

Additional file 5: Figure S2 European League Against Rheumatism (EULAR) responses of at least ‘moderate’ and ‘good’ for those patients with prior use of TNF antagonists and TNF antagonist-naive patients. Data are shown as observed values for all evaluable patients at each time point during long-term treatment with adalimumab (ADA). LO, last observation. Click here for file

Additional file 6: Figure S3 **(A)** Low disease activity (LDA) and **(B)** disease remission (REM) defined as Simplified Disease Activity Index (SDAI) ≤11 and SDAI ≤3.3, respectively, for those patients with prior use of TNF antagonists and TNF antagonist-naive patients. Percentages of patients with **(C)** minimal important difference ≥0.22 on the Health Assessment Questionnaire Disability Index (HAQ DI) and **(D)** normal function defined as HAQ DI ≤0.5. Data are shown as observed values for all evaluable patients at each time point during long-term treatment with adalimumab (ADA). LO, last observation. Click here for file
