# Supplementary material for: Safety and effectiveness of adalimumab in patients with rheumatoid arthritis over 5 years of therapy in a phase 3b and subsequent postmarketing observational study
Source: Arthritis Res Ther. 2014 Jan 27;16(1):R24. doi: 10.1186/ar4452 (PMC3979145; doi:10.1186/ar4452)
Supplement: Additional file 1 — Name and address of the Independent Ethics Committee/Institutional Review Board (IEC/IRB) from the ReAlise Study (NCT00234884). [file ar4452-S1.docx]

| **Name and Address of IEC/IRB from the ReAlise Study (NCT00234884)** |
| --- |
| Commissie Medische Ethiek – Toetsingcommissie  UZ Leuven  Herestraat 49  3000 Leuven  Belgium |
| Comitato Etico dell'A.O. "Istituti Ospitalieri" di Verona  P.le Scuro, 10  37134 Verona, Italy |
| Centro Hospitalar do Alto Minho, EPE - Comissão de Ética  Largo Conde de Bertiandos,  4990-041 Ponte de Lima, Portugal |
| Comitato Etico  Azienda Policlinico Umberto I Viale del Policlinico, 155  00185 Roma, Italy |
| Comitato Etico Indipendente Azienda Ospedallera Universitaria Policlinico S. Orsola – Malpighi di Bologna Via Albertoni, 15  40138 Bologna, Italy |
| Hosptial Militar  Principal  Comissăo de Etica  Praca da Estrela,  1249-075 Lisboa, Portugal |
| Comitato Etico  Azienda Universitaria Policlinico "G. Martino" Via Consolare Valeria  98100 Messina, Italy |
| Comité Ético de Investigación Clínica de Asturias  Secretaría del CEIC (5 planta del Centro de Rehabilitación)  'C/ Celestino Villamil, s/n  33006 Oviedo – Asturias – Spain |
| CEIC Hospital General de Castellón  'Facturación, planta baja  Avda. Benicasim, s/n  12004 Castellon |
| Research Ethics Committee Harrogate District Hospital Strayside Wing  Lancaster Park Road  Harrogate HG2 7SX, UK |
| Comitato Etico  Azienda Ospedaliera  Istituti Clinici di Perfezionamento di Milano  Via Castelvetro, 32  Milano, Italy |
| Comitato Etico ASL 3 Genovese di Genova  Via Bertani 4  16125 Genova, Italy |
| South Eastern Sydney Illawarra Area Health Service HREC - Central  Hospitals Network  Research Management Office  Level 3, Room 28  James Laws House St George Hospital Gray Street  KOGARAH NSW 2217  Australia |
| Sydney South West Area Health Service - Royal Prince Alfred  Hospital HREC  Research Development Office  Level 3, Building 92  Royal Prince Alfred Hospital Missenden Road  CAMPERDOWN NSW 2050  Australia |
| Comitato Etico per la  Sperimentazione Clinica  dei Medicinali dell' Az.Ospedaliero-Universitaria Pisana di Pisa  Via Roma, 67  56126 – Pisa - Italy |
| Cairns Base Hospital Research Ethics Committee  Cairns Hospital  PO Box 902  Cairns, QLD 4870, Australia |
| Centro Hospitalar Lisboa Ocidental, EPE - Hospital Egas Moniz  Comissão de Ética  Rua da Junqueira, 126, 1349-019 Lisboa, Portugal |
| Comitato Etico  Dell'A.O. Ospedale Di Circolo  E Fondazione Macchi Viale L Borri 57,21100 Varese Italy |
| Austin Health Human Research Ethics Committee Henry Buck Building  Austin Hospital  145 Studley Road  Heidelberg, VIC 3084 Australia |
| Comité Ético de Investigación Clínica de la Rioja (CEICLAR) Consejería de Salud Edificio CIBIR  'C/ Piqueras 98, 3ª planta  26006 – Logroño- La Rioja |
| Comitato Etico Dell'A.O. Universitaria Maggiore della Carità di Novara Corso Mazzini, 18  28100 Novara  Italy |
| Hospital Garcia de Orta, EPE  Comissão de Ética Av.Torrado da Silva,  2801-951 Almada, Portugal |
| Comitato Etico Scientifico Ospedale Niguarda Cà Granda Piazza Ospedale Maggiore, 3  20162 Milano, Italy |
| CEIC Hospital Clínic i Provincial  Servicio de Farmacia, Escalera 8 Sótano  'C/ Villarroel, 170  08036 Barcelona - Spain |
| Comitato Etico dell' Az.Ospedaliero-Universitaria  Osp. Riuniti Di Foggia  V. Le Pinto  71100 Foggia, Italy |
| Comitato Etico Azienda USL 4 Prato Piazza Ospedale 1  59100 Prato, Italy |
| Comitato di Biotica della ASL di Sassari Via Montegrappa, 82  07100 Sassari, Italy |
| CEIC Hospital Universitario Reina Sofía  'Edificio de Consultas externas planta menos 1-Sótano  'Avda. Menéndez Pidal, s/n  14004 Cordoba – Spain |
| Salford and Trafford  LREC  Salford and Trafford Health Authority  5th Floor, Peel House  Albert street  Eccles Manchester  M30 0NJ, UK |
| Comitato Etico Ospedale V. Cervelio Via Trabucco 180  90146 Palermo, Italy |
| CEIC Área 9-Hospital Severo Ochoa de Leganés  Secretaría CEIC. 1ª Planta Zona C. (Escalera de emergencia entre secretaría de RX y Hospital de Día)  'Avda. Orellana, s/n  28911 Leganes – Madrid – Spain |
| Comitato Etico dell'Azienda Ospedaliera Universitaria S.Martino di  Genova  Largo Rosanna Benzi, 10  16132 Genova  Italy |
| Spett.le Comitato Etico Aziendale Azienda Ospedaliera Universitaria Santa Maria della Misericordia  Via Colugna, 50  33100 Udine  Italy |
| Comitato Etico Unico per la Provincia di Parma  Via Gramsci, 14  43100 Parma, Italy |
| Comitato Etico per la Sperimentazione  Clinica dei Farmaci  ASL Pescara  c/o Direzione Sanitaria P.O. Pescara  Via R. Paolini, 45  65100 Pescara, Italy |
| CEIC Hospital de la Santa Creu i Sant Pau  Secretaría del Servicio de Farmacología Clínica, Antiguo archivo de historias clínicas 1er piso  'Avda. Sant Antoni Mª Claret, 167  08025 Barcelona - Spain |
| CEIC Hospital Mútua de Terrasa  'Secretaría del CEIC Plaza Dr. Roberto, 5  08221 Terrasa Barcelona Spain |
| Comitato Etico dell' Azienda Ospedaliera Istituto Ortopedico  "Gaetano Pini"  Piazza Cardinal Ferrari, 1  20122 Milano, Italy |
| CEIC Hospital Regional Universitario Carlos Haya  Comité Ético & Gestión Económica / 7ª Planta Pabellón A Avda. de Carlos Haya, s/n  29010 Malaga -Madrid,  Spain |
| Hospital do Divino Espírito Santo  Comissão de Ética  Rua da Grotinha – Arrifes,  9504-516 Ponta Delgada Açores, Portugal |
| CEIC Hospital de L Hospitalet (Consorci Sanitari Integral) Josep Molins, 29-41  08906 L Hospitalet de Llobregat - Barcelona |
| Comitato Etico Locale  Azienda Ospedaliera Universitaria Senese  Via delle Scotte, 14  53100 Siena, Italy |
| CEIC Hospital Universitario "Puerta del Mar" Departamento de Docencia, 9ª planta  'Avda. Ana de Viya, 21  11009 Cadiz - Spain |
| Comitato Etico  Aziende Sanitarie Umbria di Perugia  Via Della Rivoluzione, 16  Ellera di Corciano  06070 Perugia, Italy |
| Comitato Etico  A.O. Spedali Civili di Brescia  P. le Spedali Civili, 1  25126 Brescia, Italy |
| Harrogate Health Care NHS Trust Local Research Ethics Committee  Harrogate District Hospital Strayside Wing  Lancaster Park Road  Harrogate HG2 7SX, UK |
| Comitato Etico Azienda Ospedaliera San Gerardo  Via Pergolesi, 33  20052 Monza, (MI) Italy |
| Comitato Etico per la Sperimentazione del Farmaco della ASUR  zona territoriale d Jesi  Via Gallodoro, 68  60035 Jesi Ancona, Italy |
| Wrightington, Wigan and Leigh Local Research Ethics Committee  Wigan & Leigh NHS Trust Royal Albert Edward Infirmary Blackrock  164 Wigan Lane  Wigan WN1 2LA, UK |
| CEIC Hospital Universitario Virgen de las Nieves  'Secretaría Técnica del CEIC-Edificio Licinio de la Fuente 4ª planta.  'Dr. Azpitarte nº 4  18012 Granada - Spain |
| South Tees LREC  1st Floor, Academic Centre James' Cook University Hospital Marton Road  Middlesbrough  Cleveland, TS4 3BW, UK |
| 'CEIC Centro Médico Teknon  'Fundación Teknon, Feli Lopez  'C/Vilana,12  08022 Barcelona, Spain |
| CEIC Hospital General de Vic. Fundación de Osona per a la reserca i Educacio Sanitaries FORES  Secretaría del CEIC - Planta 0  Francesc Pla "El Vigatà," n.1  08500 Vic – Barcelona – Spain |
| Comitato Etico Independente (IEC) Segreteria Scientifica c/o Servizio Farmacia Policlinico Consorziale di Bari  P.zza G. Cesare, 11  70124 Bari, Italy |
| CEIC Corporació Sanitária del Parc Taulí  Fundació Parc Taulí - Edificio Santa Fe - Ala izquierda, 2ª planta  Fundació Parc Taulí Institut Universitari-UAB Edifici Victòria Eugènia Fundació Parc Taulí Institut Universitari-UAB Edifici Victòria Eugènia  'Parc Taulí, 1  08208 Sabadell – Barcelona - Spain |
| Hunter New England Health Research Ethics Unit  Administration Building  Lookout Road  New Lambton NSW 2305 Australia |
| 'CEIC -Área 5-Hospital Universitario La Paz  'Planta octava del hospital general, despacho 818-819  'Paseo de la Castellana, 261  28046 Madrid - Spain |
| Comitato Etico della Provincia di Modena  Via del Pozzo 71  41100 Modena, Italy |
| CEIC Área 11-Hospital 12 de Octubre  Hospital Materno Infantil, Sotano 2  Avda. de Córdoba s/n  28041 Madrid Spain |
| Comitato Etico dell' Azienda Ospedaliero-Universitaria di Cagliari  Via San Giorgio, 12  09124 Cagliari, Italy |
| CEIC Institut Municipal d´l Assitencia Sanitaria Secretaria Técnica CEIC – MAS – 1ª PLANTA Doctor Aiguader, 88 – Edificio PRBB  08033 Barcelona |
| CEIC Hospital Universitario San Cecilio  'Area de Dirección 2ª planta Izda.,  'Avda. del Dr. Oloriz, 16  18012 Granada Spain |
| CEIC Área 8-Hospital de Móstoles  'Dirección Médica 2ª Planta  'C/ Río Júcar, s/n  '28935 'Móstoles - Madrid - Spain |
| Comitato Etico Az. Osp. S. Camillo-Forlanini Circonvallazione Gianicolense, 87  00152 Roma, Italy |
| Comitato Etico  AUSL LE  Via Miglietta, 5  73100 Lecce, Italy |
| CEIC Hospital General Universitario de Elche  Unidad de Investigación; Comisión de Docencia (Biblioteca) Camí de l'Almazara,11  03203 Elche  Alicante |
| CEIC Hospital Universitario Virgen Macarena  'Unidad de Investigación - 2ª planta  'Avda. Dr. Fedriani, 3  41009 Sevilla Spain |
| Comitato Etico Indipendente (IEC)  Segreteria Scientifica c/o Servizio Farmacia Del Policlinico  Consorziale di Bari  P.zza G. Cesare, 11  70124 Bari, Italy |
| Comitato Etico  per la Sperimentazione Clinica sul Malato  Via Potito Petrone  85100 Potenza |
| CEIC Hospital de Lleida "Arnau de Vilanova"  'Recerca, Docència i Qualitat - Planta 1ª edificio antiguo del Hospital  Universitario Arnau de Vilanova  'Avda. Alcalde Rovira Roure, 80  25006 Lérida España |
| CEIC Hospital General Universitario de Alicante  'Centro de Diagnósticos- 3ª Planta (Edificio Gris)  'C/Pintor Baeza, 12  03010 Alicante |
| Comitato di Bioetica della ASL di Sassari  Via Montegrappa, 82  07100 Sassari, Italy |
| Direttore Sanitario  Azienda Ospedaliera Ordine Mauriziano di Torino Umberto I Via Magellano, 1  10128 Torino, Italy |
| Comitato Etico per la Sperimentazione dell' A.O. di Padova  Via Gustiniani, 2  35128 Padova, Italy |
| Centro Hospitalar Lisboa Norte, EPE – Hospital de Santa Maria  Comissão de Ética  Av. Prof. Egas Moniz,  1649-035 Lisboa, Portugal |
| North Western Adelaide Health Service Ethics of Human Research Committee Queen Elizabeth Hospital  Woodville Road  Woodville, SA 5011, Australia |
| 'CEIC Hospital Universitario Virgen de la Victoria  'Investigación - Unidad de Gestión de Ensayos Clínicos 1ª planta  'Campus Universitario de Teatinos, s/n (Colonia Santa Inés)  29010 Málaga Spain |
| 'CEIC Área 10- Hospital Universitario de Getafe  'Secretaría del CEIC, Unidad de Investigación  Ctra. De Toledo, Km 12,500  28905 Getafe Madrid |
| 'CEIC Hospital Universitario Dr. Peset  'Secretaría del CEIC, 1ª Planta, Consultas Externas  'Juan de Garay, 21  46017 Valencia Spain |
| CEIC Hospital Ciudad de Jaén  'Unidad de Investigación - Antigua Escuela de Enfermeras Planta baja  'Avda. del Ejercito Español, 10  23007 Jaén Spain |
| Comitato Etico  ASL Locale n. 3 Genovese  Via G. Maggio, 6  16147 Genova, Italy |
| Comitato Etico Ospedale S. Raffaele Via Olgettina, 60  20132 Milano, Italy |
| Comitato Etico Provinciale di Reggio Emilia  V.le Risorgimento, 57  42100 - Reggio Emilia- ITALY |
| Comitato Etico  Azienda Ospedaliera di Vimercate  Viale C. Battisti, 23  20059 Vimercate (MI) Italy |
| Hospitais da Universidade de Coimbra (HUC) Comissão de Ética  Av. Bissaya Barreto,  3000-075 Coimbra, Portugal |
| Comitato Etico  Azienda Ospedaliera (Gaetano Rummo" Via Dell' Angelo, 1  82100 Benevento, Italy |
| Sir Charles Gairdner Hospital Ethics Committee  Sir Charles Gairdner Hospital  Verdun Street  Nedlands, 6009WA, Australia |
| Comitato Etico Indipendente(IEC)  Segreteria Scientifica c/o Servizio Farmacia Del Policlinico  Consorziale di Bari  P.zza G. Cesare, 11  70124 Bari, Italy |
| Comitato Etico Azienda Ospedaliera "Maggiore della Carità" di  Novara  Corso Mazzini n. 18  28100 Novara  Italy |
| Comitato Etico dell' Azienda Ospedaliera Universitaria Policlinico  "P. Giaccone" dell' Università degli Studi di Palermo  Via del Vespro, 129  90127 - Palermo  Italy |
| Comitato Etico  della Provincia di Ferrara  C.so Giovecca 203  44100 Ferra, Italy |
| Presidente del Comitato Etico  dell' Azienda Ospedaliera della Seconda Università degli Studi di  Napoli  Via Costantinopoli, 104  80138 - Napoli  Italy |
| Comitato Etico  dell' Azienda Policlinico Umberto I Viale del Policlinico, 155  Roma, Italy |
| CEIC Hospital Universitario de Bellvitge  'Secretaría Administrativa - Edifici Unitat de Recerca  'C/ Feixa Llarga s/n  '08907 ' - L´Hospitalet de Llobregat – Barcelona - Spain |
| Instituto Português de Reumatologia  Comissão de Ética  Rua da Beneficência, 7, 1050-034 Lisboa, Portugal |
| Princess Alexandra Hospital Human Research Ethic Committee  Ipswich Road  Woolloongabba, QLD 4102, Australia |
| Brighton LREC  Brighton and Hove PCT  6th floor, Vantage Point New England Road Brighton  BN1 4GW, UK |
| Oxford Research Ethics Committee  John Radcliffe Hospital Research & Development Room 13  1st Floor, Manor House  Oxford OX3 9DZ, UK |
| 'CEIC Área 8 Fundación Hospital Alcorcón  'Secretaría del Comité Ético de Investigación Clínica (Planta Sótano)  'C/ Budapest, 1  28922 Alcorcon-Madrid - Spain |
| CEIC Área 4-Hospital Universitario Ramón y Cajal  'Planta menos 2 Drcha.  'Ctra. de Colmenar Viejo, Km. 9,1  28034 Madrid Spain |
| CEIC Autonómico de ensayos clínico de Andalucía  'Consejería de Salud. Planta baja  'Avda. Innovación s/n. Edif. Arena 1.  41020 Sevilla, Spain |
