# Supplementary material for: Safety and effectiveness of adalimumab in patients with rheumatoid arthritis over 5 years of therapy in a phase 3b and subsequent postmarketing observational study
Source: Arthritis Res Ther. 2014 Jan 27;16(1):R24. doi: 10.1186/ar4452 (PMC3979145; doi:10.1186/ar4452)
Supplement: Additional file 2 — Name and address of the Independent Ethics Committee/Institutional Review Board (IEC/IRB) from the ReAct Study (NCT00448383). [file ar4452-S2.docx]

| **Name and Address of IEC/ IRB from the ReAct Study (NCT00448383)** |
| --- |
| SESAHS (South Eastern Sydney Area Health Service) Ethics Committee St. George Hospital  Gray Street  Kogarah NSW 2217, Australia |
| Ethics Review Committee RPAH Zone  Royal Prince Alfred Hospital Research Development Office Level 5, Building 72  59 Missenden Road  Camperdown NSW 2050, Australia |
| Cairns Base Hospital Research Ethics Committee  Cairns Hospital  PO Box 902  Cairns, QLD 4870, Australia |
| Austin Health Human Research Ethics Committee  Research Support Unit, North Wing, Heidelberg Repatriation Campus  300 Waterdale Road  Heidelbert West, VIC 3081, Australia |
| St. Vincents Hospital Research Ethics Committee  St. Vincents Hospital  Victoria Street  Darlinghurst NSW 2010, Australia |
| Royal Hobart Hospital Ethics Committee Office for Research, University of Tasmania  GPO Box 252-1  Hobart Tasmania 7000, Australia |
| Hunter Health Human Area Research Ethics Committee  Royal Newcastle Hospital  Newcastle, NSW 2300, Australia |

| Melbourne Health Research Directorate  The Royal Melbourne Hospital Parkville  VIC 3050, Australia |
| --- |
| Royal Brisbane and Women's Hospital and  Health Service District |
| North Western Adelaide Health Service Ethics of Human Research Committee Queen Elizabeth Hospital  Woodville Road  Woodville, SA 5011, Australia |
| Sir Charles Gairdner Hospital Ethics Committee  Sir Charles Gairdner Hospital  Verdun Street  Nedlands, 6009 WA, Australia |
| Princess Alexandra Hospital Human Research  Ethics Committee  Ipswich Road  Woolloongabba, QLD 4102, Australia |
| Ethics Committee of the General Hospital Linz  Krankenhausstrasse 9  4020 Linz, Austria |
| Ethics Committee of the City of Vienna  Schottenring 24  1010 Wien, Austria |
| Ethics Committee of the County of Lower Austria  Department for Medical and Hospital Law  Landhausplatz 1, Building 15b  3109 St. Polten, Austria |

| Ethics Committee of the County of Vorarlberg  Rathausstrasse  5801 Bregenz, Austria |
| --- |
| Ethics Committee of the County of Carinthia  Hospital Klagenfurt  St. Veiter Strasse 47  9026 Klagenfurt, Austria |
| Ethics Committee of the County of Carinthia  Landeskrankenhaus Klagenfurt  St. Veiter Strasse 47  9026 Klagenfurt, Austria |
| Ethics Committee of the Medical Faculty of the University of Innsbruck  Innrain 43  A-6020 Innsbruck, Austria |
| Ethikkommission des Landes Salzburg Amt der Salzburger Landesregierung  Sebastian-Stief-Gasse 2  5010 Salsburg, Austria |
| Ethics Committee of the Medical Faculty of the University of Graz  Auenbruggerplatz 29  8036 Graz, Austria |
| Ethics Committee of the Medical Faculty of the  University of Vienna  Spitalgasse 23  1090 Wien, Austria |
| Ethics Committee of the County of Upper Austria  Hospital Wagner-Jauregg  Wagner-Jauregg-weg 15  4020 Linz, Austria |
| Ethics Committee of the Convent Hospital of the  Hospitallers Graz-Eggenberg  Bergstarsse 27  8020 Graz, Austria |

| Ethics Committee of the Hospital Oberpullendorf  Spitalstrasse 32  7350 Oberpullendorf, Austria |
| --- |
| ULB Erasme Ethics Committee  Route de Lennik 808  1070 Andrelecht, Belgium |
| EC Hopital Louis Caty  Rue Louis Caty 136  7331 Baudour, Belgium |
| Comite d'Ethique Clinique  Notre-Dame de Grace  Nivelles 22  6042 Gosselies, Belgium |
| Comite d'Ethique  Center Hospitalier  De Jolimont-Lobbes, Belgium |
| EC – OCWM Antwerpen  Lange Gasthuisstraat 35-37  2000 Antwerpen, Belgium |
| Ethics Committee UZA  Wilrijkstraat 10  2650 Edegem, Belgium |
| EC Maria Midellares CP St. Niklaas  Hospitaalstraat 17  9100 Sint-Niklaas, Belgium |
| Commission d'Ethique Biomedicale  Hospital Facultaike  Belgium |

| Commission de Bioethique  De la Defense  ACOS WB Quarter Reine Artrid rue Bruyn  1120 Bruxelles, Belgium |
| --- |
| Amburgs Universitair Centrum Committee Medisch Ethiek Universitair Campus  3590 Depenbeek, Belgium |
| Ethisch Commite  AZ Groeninge, Belgium |
| Committee de'Ethique  Hospital Francais-Cesar de Paepe, Belgium |
| Committee de'Ethique  Centre de Sante des Fagnes, Belgium |
| Ethische commissie AZ Maria Middelares, Belgium |
| Clinique St. Elisabeth  Brussels, Belgium |
| Ziekenhuis Aalst  Merestraat 80  9300 Aalst, Belgium |
| Ethische Committee  AZ St. Jan  Belgium |
| EC Institut Jules Bordet  Rue Heger Bordet 7  1000, Bruxelles, Belgium |
| Committee de'Ethique  Hospitalier UCL, Belgium |

| EC – Hopital Amboise Pare  Boulevard Kennedy 2  7000 Mons, Belgium |
| --- |
| EC – AZ  Damiaan Gouwelozestraat 100  8400 Oostende, Belgium |
| EC – AZ St. Lucas/St. Jozef  Sint Lucaslaan 29  8310 Assebroek, Belgium |
| Faculty of Medicine, Ethics Committee  University of Liege  Domaine Universitaire Sart-Tilman  4000 Liege, Belgium |
| Comite de Ethique  Centre Hospitalier Regional  St. Joseph, Belgium |
| EC – Medisch Instituut St. Augustinus  Oosterveldlaan 24  2160 Wilrijk, Belgium |
| Hopitaux Iris SUD  1050 Bruxelles,Belgium |
| EC – Europaziekenhuizen  Avenue Defre 206  1180 Uccle, Belgium |
| EC – Clinique Reine Fabiola  Du Centenaire 73  6061 Montingnies-sur-Sambre, Belgium |
| EC – Hopital Brugmann  Place Van Gehuchten 4  1090 Bruxelles, Belgium |

| Elisabeth Ziekenhuis  Gentse Steenweg 132  8340 Sysele, Belgium |
| --- |
| Centre Hospitalier InterRegional Edith Cavell  Rue Wayez 35  1420 Braine-I'Alleud, Belgium |
| Committee d'Ethique  CHU de Charleroi, Belgium |
| EC – CHU Tivoli  Avenue Max Buset 34  7100 La Louviere, Belgium |
| EC – AZ Sint Jozef  Steenweg op Merksplas 44  2300 Turnhout, Belgium |
| EC – Hopital St. Pierre  Rue Reine Babiola 9  1340 Ottignies, Belgium |
| CCPPRB Paris – Pitie Salpetriere Hopital de la Pitié – Salpetrière Pavillion Jacquart  47 Boulevard de l'hopital  75651, Paris, France |
| EC – Heilig Hart Kliniek  Roeselaere Wilgenstraat 2  8000 Roeselaere, Belgium |
| Commissie Medische  Ethiek VUB, Belgium |
| EC – Katholieken Universiteit Leuven  Herestraat 49  3000 Leuven, Belgium |

| Ethik-Kommission bei der LAK Berlin  Flottenstr. 28-42  13407 Berlin, Germany |
| --- |
| Ethik-Kommission der Brandenburgischen Landesarztekammer  Dreifertstr. 12  03044 Cottbus, Germany |
| Ethik-Kommission der Arztekammer Sachsen-Anhalt  Am Kirchtor 9  06108 Halle, Germany |
| Ethik-Kommission bei der LAK Baden-Wurttemberg  Jahnstrasse 40  70596 Stuttgart, Germany |
| Ethik-Kommission bai der LAK Rheinland-Pfalz  Deutschhausplatz 3  55116 Mainz, Germany |
| Ethik-Kommission der Justus-Liebig Universitat Giessen  Gaffkystr. 11c  35385 Giessen, Germany |
| Ethik-Kommission I der Medizinischen Fakultat Heidelberg  Bergheimer Str. 58  69115 Heidelberg, Germany |
| Ethik-Kommission an der Medizinischen Fakultat der RWTH Aachen  Pauweisstr. 30  52057 Aachen, Germany |
| EthikKommission der Arztekammer Schleswig-Holstein  Bismarckallee 8-12  23795 Bad Segeberg, Germany |

| Ethik-Kommission des Landes Bremen  Zentralkrankenhaus Sankt-Jurgen-Strasse  Sankt-Jurgen-Strasse 1  28205 Bremen, Germany |
| --- |
| Ethik-Kommission der Medizinschen Fakultat der Westfalischen Wilhelms- Universitat Munster und der Arztekammer Westfalen-Lippe  Waldeyerstr. 27  48149 Munster, Germany |
| Ethik-Kommission des Universitatsklinikum Campus Charite Mitte  Schumannstrasse 20-21  10098 Berlin, Germany |
| Ethik-Kommission der Sachsischen Landesarztekammer  Schutzenhohe 16  01074 Dresden, Germany |
| Ethik-Kommission der Landesarztekammer Baden-Wurttemberg  Jahnstr. 40  70597 Stuttgart, Germany |
| Ethik-Kommission der Bayerischen Landesarztekammer  Muhlbaurstr. 16  81677 Munchen, Germany |
| Ethik-Kommission der LAK Nordrhein  Tersteegenstr. 31  40474 Dusseldorf, Germany |
| Ethik-Kommission der AK Berlin  Flottenstr. 28-42  13407 Berlin-Reinickendorf, Germany |
| Ethik-Kommission der Medizinischen Fakultat der Universitat Leipzig  Hartelstrasse 16-18  04107 Leipzig, Germany |
| Ethik-Kommission der LAK Hamburg  Heinrich-Hertz-Str. 125  22083 Hamburg, Germany |

| Ethik-Kommission der Friedrich-Schiller Universitat Jena an der Medizinischen Fakultat  Dornburgerstr. 159  07740 Jena, Germany |
| --- |
| Ethik-Kommission der Med. Fakultat Der Universitat zu Lubeck Ratzeburger Str. 160  23538 Lubeck, Germany |
| Ethik-Kommission der Sachischen  Landesartzekammer Geschaftsstelle der Sachsischen Landesarztekammer  Schutzenhohe 16  01099 Dresden, Germany |
| Ethik-Kommission der Medizinischen Fakultat der Universitat Erlangen-Nurnberg  Universitatsstr. 40  91054 Erlangen, Germany |
| Ethik-Kommission der J.-W. Goethe Universitat Frankfurt  Theodor-Stern-Kai 7  60590 Frankfurt, Germany |
| Ethik-Kommission der Landesarztekammer Hessen  Im Vogelsang 3  60488 Frankfurt, Germany |
| Ethik-Kommission der Medizinischen Fakultat der Otto-von Guericke-Universitat Magdeburg  Leipziger Str. 44  39120 Magdeburg, Germany |
| Ethik-Kommission an der Medizinischen Fakultat der Eberhard-Karls-Universitat  Keplerstr. 15  72074 Tubingen, Germany |
| Ethik-Kommission der Artzekammer Berlin  Flottenstr. 28/42  13407 Berlin, Germany |
| Ethik-Kommission bei der LAK Hessen  Im Vogelsgesang 3  60488 Frankfurt, Germany |
| Ethik-Kommission bei der LAK Bayern  Muhlbaurstrasse 18  81677 Munchen, Germany |

| Ethik-Kommission der Arztekammer Niedersachsen  Berliner Allee 20  30175 Hannover, Germany |
| --- |
| Ethikkommission der Universistat Wurzburg  Versbacher str. 3  97080 Wurzburg, Germany |
| Ethik-Kommission bei der AK Mecklenburg- Vorpommern  Humboldstr. 6  18055 Rostock, Germany |
| Ethik-Kommission der Arztekammer Nordrhein  Tersteegenstr. 31  40474 Dusseldorf, Germany |
| Ethik-Kommission der MHH  Carl-Neuberg-Strasse 1  30625 Hannover, Germany |
| Ethik-Kommission der Medizinischen Fakultat der Ruhr-Universitat Bochum  Universitatsstr. 150  44801 Bochum, Germany |
| Ethik-Kommission des katholischen Krankenhaus der Klinik Essen Sud  Propsteistrasse 2  45239 Essen, Germany |

| Ethik-Kommission an der Georg-August- Universitat, Medizinische Fakultat  Robert-Koch-Str. 40  37075 Gottingen, Germany |
| --- |
| Klinikum der Universitat Regensburg  Ethik-Kommission am Zentrum fur klinische Studien  93042 Regensburg, Germany |
| Ethik-Kommission bei der Arztekammer Mecklenburg-Vorpommern  Humboldtstr. 6  18055 Rostock, Germany |
| Ethikkommission der Sachsischen Landesartzekammer  Schutzenhohe 16  01099 Dresden, Germany |
| Ethik-Kommission Albert-Ludwigs-Universitat Freiburg  Elsasser Str. 2m, Haus 1 A  79110 Freiburg, Germany |
| Ethik-Kommission der Medizinischen Fakutat der Universitat zu Koln  Josef-Stelzmann-Str. 9  50931 Koln, Germany |
| Ethik-Kommission der Medizinischen Fakultat der Henirich-Heine Universitat  Moorenstrasse 5  40225 Dusseldorf, Germany |
| Ethik-Kommission der Medizinischen Fakultat Carl Gustav Carus der Technischen Universitat Dresden  Fetscherstrasse 74  01307 Dresden, Germany |
| Ethik-Kommission der Christian-Albrechts- Universitat zu Kiel  Schwanenweg 20  24105 Kiel, Germany |
| Ethik-Kommission der BFU Berlin  Hindenburgdamm 30  12200 Berlin, Germany |

| A'Propaeudeutic Medicine Dpt. Regional General Ippokrateio Hospital  Konstantinoupoleos 49  Thessaloniki 546 42, Greece |
| --- |
| General University Hospital of Patras  Ethics Committee  26500 Patras, Greece |
| General Hospital of Athens  "Sismanogleio Ethics Committee  15100 Athens, Greece |
| University General Hospital of Heraklion Ethics Committee Stavrakia  81352, Heraklion, Greece |
| General Hospital Laiko Ethics Committee  17, Ag. Thoma,  11527 Athens, Greece |
| General University Hospital of  Ioannina Ethical Committee  45500 Ioannina, Greece |
| 401 Army Hospital of Athens  Ethical Committee  11525 Athens, Greece |
| General Hospital of Athens  "Evagelismos" Ethical Committee  45-47 Ipsilandou Street  11521 Athens, Greece |
| NI.M.T.S. Hospital Ethical Committee  10 Monis Petraki Street  11521 Athens, Greece |
| IRB/IEC of G.P.N. Th. "Papanikalaou" Asvestoxori/Ehoxi  TK 57010 Thesaloniki, Greece |
| General Hospital K.A.T. Ethics Committee  2, Nikis Street  14500 Athens, Greece |
| G.P.N.A. "G. Gennimatas" Ethics Committee  Mesogeion Ave. 154  11527 Athens, Greece |

| General University Hospital of Larissa  Ethics Committee  41110 Larissa, Greece |
| --- |
| AHEPA General Hospital of  Thessaloniki Ethics Committee  54636 Thessaloniki, Greece |
| Ethical Committee  General Hospital Laiko  17 Ag. Thoma  11527 Athens, Greece |
| Euroclinic Ethics Committee  9 Athanasiadou Street  11521 Athens, Greece |
| Navy Hospital of Athens Ethical Committee  70 Dinokratous  11521 Athens, Greece |
| 3rd IKA Hospital, Ethical Committee  4 Kapodistriou Street  10682 Athens, Greece |
| General Hospital of Athens  "Laiko" Ethics Committee  11527 Athens, Greece |
| Asclepieion General Hospital  Ethical Committee  16600 Voula, Greece |
| A General Hospital of Thessaloniki 'Agios  Pavlos' Ethics Committee  54625 Thessaloniki, Greece |
| Comitato Etico Dell'A.O. DiVerona – Policlinico 9B Rossi Ple Sevro 10 Verona, Italy |
| Comita to Etico  Azienda Policlinico Umberto I Viale del Policlinico, 15S  00161 Roma, Italy |

| Comitato Etico  Azienda Ospedallera Di Bologna  Policlinico S. Orsola – Macpighi  C/o Direzione Ospedallera via Masserenti, 9  40138 Bologna, Italy |
| --- |
| Comitato Etico  Azienda Universitaria Policlinico "G. Martino" Via Consolare Valeria  98100 Messina, Italy |
| Comitato Etico Dell'Azienda Ospedaliera DiVerona Policlinico G.B. Rossi  P. LeScuro  32136 Verona, Italy |
| Comitato Etico AVSL 3 Genovese  Via G. Maggio 6  16147 Genova, Italy |
| Comitato di Bioetica  Azienda Ospedaliera Pisana  53100 Pisa, Italy |
| Comitato Etico Azienda Ospedaliera San Gerardo Di Monza  Via Donizetti 10E  20052 Monza, Italy |
| Comitato Etico  Dell'A.O. Ospedale Di Circolo  E Fondazione Macchi di Verese, Italy |
| Ethical Committee Regione Piemonte  Corso Retina Margherita 153 BIS  10122 Torino, Italy |
| Comitato Etico  Ospedale Maggiore Ca Granda  Piazza Ospedale Maggiore 3  20162 Milano, Italy |
| Comitato Etico Degli Osp. Riuniti Di Foggia  V. Le Pinto  71100 Foggia, Italy |

| Comitato Etico Ospedale di Prato  Azienda ASL 3 Prato Piazza Ospedale 1  59100 Prato, Italy |
| --- |
| Comitato Etico  Azienda ASL N.1 Sassari  Via Montegrappa, 82  07100 Sassari, Italy |
| Comitato Etico  Dell. A.O. Polo Universitario "L. Sacco"  Via G.B. Grassi 76  Milano, Italy |
| Comitato Etico  Der La Sperimentazione Clinica de Farmaci  Azienda Ospedaliere Careggi, Italy |
| Comitato Etico DIMI Viole Benedetto XV, 6  16132 Genova, Italy |
| Comitato Etico  Policlinico Universitario  P. Le S.M. Della Misericordia 1  33100 Udine, Italy |
| Comitato Etico per la Sperimentazione  Clinica dei Farmaci  ASL Pescara  Presidio Ospedaliero "Spirito Santo" ASL Pescara  Via Fonte Romana  65100 Pescara, Italy |
| Comitato Etico Dell Instituto Orthpedico  "Li Pini"  P. 22A Cardinal Ferrari, 1  20122 Milano, Italy |
| C.E. – AUSL Bologna Citta  C/o Farmacia Ospedale Maggiore Bologna  Largo Nigrisoli, 2, Bologna, Italy |
| Comitato Etico Ospedale Cervelio  Via Trabucco 180  80160 Palermo, Italy |

| Comitato Etico  Aziende Sanitarie Umbria  Via Cotani 21/I  06126 Perugia, Italy |
| --- |
| Comitato Etico  A.O. Spedali Civili  P. Le Ospedale 1 Brescia, Italy |
| Comitato Etico ASLS  Via Gianporo 6P  60035 Jeli, Italy |
| Comitato Etico  Azienda Sanitaria N2 Castrovillari  Via degli Ospedali 1  87012 Castrovillari (CS), Italy |
| Comitato Etico Independence (IEC) C/o Policlinico  P.G. Cesare, 11  70126 Bari, Italy |
| Comitato Etico Independente  ASL delle Provincia di Milano 2  Via VIII Giugno, 69  20077 Melegnano (MI), Italy |
| Comitato Etico Indipendente Azienda Ospedaliera di Parma Via Gramsci, 14  43100 Parma, Italy |
| Comitato Etico Ospedole  S. Paolo  Milano, Italy |
| Comitato Etico Dell-Azienda Ospedallera "Instituto Ortopedico G. Pini  Piazzi C. Ferrari 1  Milano, Italy |
| Ethics Committee  San Carlo Hospital  Contrada Macchia Romana  85100 Potenza, Italy |

| Azienda Ospedaliera Senese Universita degli Studi di Siena Facolta di Medicina e Chirurgia  Comitato Etico Locale per Sperimentazione  Clinica del Medicinali  Strada delle Scotte  53100 Siena, Italy |
| --- |
| Comitato Etico del Policlinico de Modena  Via del Pozzo 71  41100 Modena, Italy |
| Comitato Etico Policlinico Universitario  Via San Giorgio, 12  09100 Cagliari, Italy |
| Comitato Etico ULSS 12 Vezueziana  Piazzalo S. Lorenzo Giustiniani 11/D  2E Larino-Venezia 30174, Italy |
| Comitato Etico AZ. Osp. S. Carillo-Forlanini Circonvallazione  Gianicolense 87  00152 Roma, Italy |
| Commissione Consultiva per le  Sperimentazione  Cliniche delle Regione Piemonte  C.So. Regina Margherita, 153, BIS  10122 Torino, Italy |
| Comitato di Bioetica  IRCCS Policlinico S. Matteo  P. Le Golgi, 2  27100 Pavia, Italy |
| Comitato Etico  Dell'Azienda Ospededalliera "Vito Fazzi" IECCE P. ZZA Filippo Murafone  73100 Lecce, Italy |
| Comitato Etico  Universita Studi Napoli Federico II  Via Pansini, 5  80131 Napoli, Italy |

|  |
| --- |

| Comitato Etico  Ospedale S. Raffaele  Via Olgettina, 60  20132 Milano, Italy |
| --- |
| Comitato Etico Azienda Ospedaliera Espedale Civile  Via C. Battisti, 2D Vimercate, Milano, Italy |
| Ospedale S. Gerardo  Comitato Etico  Via Donizze Hi, 106  20052 Monza, (MI) Italy |
| Comitato Etico  Azienda Ospedaliera (Gaetano Rummo" Via Dell'Angelo, 1  82100 Benevento, Italy |
| Comitato Etico per la Sperimentazione  Via Gustiniani 2  Padova, Italy |
| Comitato Etico Indipendente di riferimento Individuato dall Retione Piemonte Assessorato alla Sanita  Corso Regina Margherita, 153./BIS  10122 Torino (To), Italy |
| Comitato Etico  Policlinico di Medicina Interna a Geriatria  Via del Vespro 141  90127 Palermo, Italy |
| Comitato Etico  Azienda Ospedaliera Arcispedale S. Anna  Divisione di Reumatologia  C.so Giovecca 203  44100 Ferra, Italy |
| Direzione Sanitaria  Viale Risorgimento 80  42100 Reggio Emilia, Italy |

| Comitato Etico  Seconda Universita degli Studi di Napoli  P. zza Miraglia  Napoli, Italy |
| --- |
| IEC  Comitato Etico  Deli Azienda Policlinico Umberto I Roma, Italy |
| Comitato di Etico Ospedale S. Paolo Localita Valloria Padiglione Vigiola  17100 Savona, Italy |
| Comitato Etico Dell'Universita Cattiolico del Sacro Cuore Policlinico Gemelli  Cargo Gemelli, 8  00168 Roma, Italy |
| Erasmus Medical Center  Medisch Etische Toetsings  Commissie  Dr. Molewaterplein 40  3015 GD Rotterdam, Netherlands |
| Universitair Medisch Centrum Utrecht Medisch Ethische Toetsingscommissie  Heidelberglaan 100  3584 CX Utrecht, Netherlands |
| T'Lage Land Ziekenhuis  METC  Postbus 3015  2700 KJ Zoetermeer, Netherlands |
| Toetsingscommissee Patient gebonden  Ondersoch  Henri Durantweg 2  8934 AD Leeuwarden, Netherlands |

| Commissie Medische Ethiek Zeeland  Oosterscheldeziekenhuis, s'Gravenpolderseweg 114  Postbus 106, 4460 BB Goes  Netherlands |
| --- |
| Medisch Centrum Rijnmond Suid, lokatie Zuider METC  Postbus 9119  3007 AC Rotterdam, Netherlands |
| Jeroen Bosch Ziekenhuis  Secretariaat METC Noord-Oost Brabant  Postbus 90153  5200 ME 's-Hertogenbosch, Netherlands |
| Atrium Medisch Centrum  Medisch Etische Toetsingscommissie  P/A BWO Gebouw V Postbus 4446  6401 CX Heerlen, Netherlands |
| Rijnstate Ziekenhuis  Medisch Ethische Toetsingscommissie  Wagnerlaan 55  6815 AD Arnhem, Netherlands |
| Viecuri Medisch Centrum  METC Gegelsweg 210  5912 BL Venlo, Netherlands |
| Medisch Centrum Alkmaar  Medisch Ethische Toetsingscommissie  Wilhelminalaan 12  1815 JD Alkmaar, Netherlands |
| Ziekenhuis Leyenburg  METC  Leyweg 275  2545 CH Den Haag, Netherlands |

| St. Ziekenhuis Lievensberg  Boerhaaveplein 1  4624 VT Bergen op Zoom, Netherlands |
| --- |
| Amphia Ziekenhuis, locatie Molengracht  METC West-Brabant  Molengracht 21  4818 CK Breda, Netherlands |
| Meander Medisch Centrum  Commissie Toetsing Wetenschappellijk  Onderzoek Ultrechtswweg 160  3818 ES Amersfoort, Netherlands |
| Medisch Ethische Toetsingscommissie  Ariensplein 1  7511 JX Enschede, Netherlands |
| Board of Directors Sint Maartenskliniek  Hengstdal 3  6522 JV Nijmegen, Netherlands |
| Vlietland Ziekenhuis, locatie Vlaardingen  METC  Holysingel 3  3136 LA Vlaardingen, Netherlands |
| Maaslandziekenhuis  Medisch Etische Toetsinscommissie  Walramstraat 23  6131 BK Sittard, Netherlands |

| Medisch Ethische Toetsingscommissie Academisch Ziekenhuis Groningen  Hanzeplein 1  9713 GZ Groningen, Netherlands |
| --- |
| Flevoziekenhuis  Medisch Ethische Toetsingscommissie  Hospitaalweg 1  1315 RA Almere, Netherlands |
| Gemini Ziekenhuis  Medisch Ethische Toetsingscommissie  Huisduinerweg 3  GZ Den Helder, Netherlands |
| Ziekenhuis Bronovo Raad van Bestuur  Bronovalaan 5  2597 AX Den Haag, Netherlands |
| Isala Klinieken, loc. Weezenlanden Medisch Ethische Toetsingscommissie  Groot Weezenland 20  8011 JW Zwolle, Netherlands |
| Ethical Committee of Centro Hospitalar do Alto  Minho – Delegacao de Ponte de Lima  Largo Conde de Bertiandos  4990-041 Ponte de Lima, Portugal |
| Ethical Committee of Hospital Militar Principal  Praca da Estrela  1200 Lisboa, Portugal |
| Ethical Committee Hospital Egas Moniz-Servico de Reumatologia  Rua da Junqueira No. 126  1349 Lisboa, Portugal |

| Ethical Committee of Hospital Garcia de Orta  Bairro do Matadouro – Pragal  2800-525 Almada, Portugal |
| --- |
| Ethical Committee of Hospital do Divino Espirito Santo  Rua da Grotinha – Arrifes  9500-370 Ponta Delgada  Acores, Portugal |
| Ethical Committee of Hospitais da Universidade de Coimbra  Av. Bissaya Barreto  3000-075 Coimbra, Portugal |
| Ethical Committee of Hospital de Santa Maria  Medicina IV C – Nucleo de Reumatologia  Av. Prof. Egas Moniz  1649-035 Lisboa, Portugal |
| Ethical Committee of Centro Hospitalar do Funchal  Av. Luis de Camoes  9004-514 Funchal  Madeira, Portugal |
| Ethical Committee of Instituto Portugues de Reumatologia  Rua da Beneficencia 7  1050-034 Lisboa, Portugal |
| Ethical Committee of Hospital de Sao Joao  Alameda Prof. Hernani Monteiro  4200-319 Porto, Portugal |
| Comite Etico de Ensayos clinicos  Hospital General Yague Servicio de Reumatologia  Avda. Del Cid Campeador, 96  09005 Burgos, Spain |

| Comite Etico de Investigacion Clinica  Hospital de Cruces  Plaza de Cruces no. 12  48903 Cruces-Barakaldo, Spain |
| --- |
| Comite Etico de Ensayos Clincos Hospital del Rio Hortega C/Cardenal Torquemada, s.n  47010 Valladolid, Spain |
| Comite Etico de Ensayos clinicos  Hospital Civil de Basurto  Avda. Montevideo, 18  48013 Bilbao, Spain |
| Comite Etico de Investigacion Clinica Hospital Clinico Universitario Lozano Blesa  Avenida San Juan Bosco, 15  50009 Zaragoza, Spain |
| Comite Etico de Ensayos clinicos  Hospital Central de Asturias  5 planta Centro de Rehabilitacion c/ Celestino Villamil, s/n  33006 Oviedo, Spain |
| Comite Etico de Investigacion Clinica  Hospital General de Castellon  Avda. Benicasin, s/n  12004 Castellon, Spain |
| Ethics Regional Committee of CAM  C/ Odonell, 50 pta 1a  28009 Madrid, Spain |
| Comite Etico de la Rioja  Spain |
| Comite Etico de Investigacion Clinica  Hospital Comarcal Sierrallana  Barrio Ganzo s/n  39300 Torrelavega, Cantabria, Spain |

| Comite Etico de Ensayos clinicos Hospital Clinico de Barcelona  Villarroel, 170  08036 Barcelona, Spain |
| --- |
| Comite Etico del Hospital Infanta Cristina  Avda. De Elvas, s/n  06080 Badajoz, Spain |
| Comite Etico del Hospital  Obispo Polanco, Spain |
| Comite Etico de Investigacion Clinica Hospital Universitario Reina Sofia  Avda. Menendez Pidal, s/n  14004 Cordoba, Spain |
| Comite Etico de Ensayos clinicos Hospital de Santa Creu i Sant Pau  Avda. Sant Antoni M Claret, 167  08025 Barcelona, Spain |
| Comite Etico del Hospital Severo Ochoa  C/ Budapest, 1  Leganes – Madrid, Spain |
| Comite Etico de Ensayos clinicos Hospital Universitario de Salamanca  Paseo de San vincente, 58-182  37007 Salamanca, Spain |
| Comite Etico del Hospital U. de Canarias  Spain |
| Comite Etico del Hospital U. Virgen  De la Victoria  Campus de Teatinos, s/n  29010 Malaga, Spain |
| Comite Etico de Ensayos clinicos  Hospital de Galdakao  10 planta Secretaria del centro de Farmacovigilancia de Pais Vasco Barrio Labeaga s/n  48960 Galdakao Vizcaya, Spain |

| Comite Etico de Ensayos clinicos Hospital General Rio Carrion  Avda. Ponce de Leon, s/n  34005 Palencia, Spain |
| --- |
| Comite Etico de Investigacion Clinica Hospital Universitario Puerta del Mar  Avda. Ana de Viya, 21  11009 Cadiz, Spain |
| Comite Etico del Hospital San Pedro De Alcantara  Spain |
| Comite Etico de Ensayos clinicos  Hospital Mutua de Terrassa  Plaza Dr. Robert, 5  08221 Tarrasa (Barcelona), Spain |
| Comite Etico de Ensayos clinicos Hospital Virgen de las Nieves  Avda. De las Fuerzas Armadas, 2  18014 Granada, Spain |
| Comite Regional de la Comunidad de Madrid  Unidad de Bioetica  Servicio de Regulacion Sanitaria 4 planta  C/ Aduanas 29  28013 Madrid, Spain |
| Comite Etico de Investigacion Clinica  Hospital General de Vic  C/ Frances Pla "El Vigata", 1  08500 VIC, Spain |
| Comite Etico de la Comunidad de Madrid Unidad de Bioetica. Servicio de Regulacion Sanitaria  C/ Aduanas, 29, Cuarta Planta  28013 Madrid, Spain |
| Comite Etico de Investigacion Clinica Consorcio Hospitalario del Parc Tauli  Parc Tauli, s/n  08208 Sabadell, Spain |

| Comite Etico de Ensayos clinicos Hospital Universitario Mugel Servet  P Isabel la Catolica 1-3  50009 Zaragoza, Spain |
| --- |
| Comite Etico de Investigacion Clinica  Del Hisoutak de Valme  Crta. Sevilla-Cadiz, s/n  41014 Sevilla, Spain |
| Comite Etico de Investicacion Clinica  Hospital Virgen de la Arrixaca  Ctra. Madrid, Cartagena s/n  30120 El Palmar – Murcia, Spain |
| Ethics Regional Committee of CAM C/Odonell, 50 pta 1  28009 Madrid, Spain |
| Comite Etico del Hospital 12 de Octubre  Avda. De Andalucia km 404  28041 Madrid, Spain |
| CEIC – IMAS  Instituto Municipal de Investigacion Medica  (IMIM)  C/ Dr. Aiguader, 80  08003 Barcelona, Spain |
| Comite Etico de Investigacion Clinica  Hospital Gregorio Maranon  C/ Doctor Esquerdo, 46  28007 Madrid, Spain |
| Comite Etico de Investigacion Clinica  Clinica Puerta de Hierro  Servicio de Farmacia  C/ San Martin de Porres, 4  28035 Madrid, Spain |
| Ethics Committee of Centro Medico Teknon  C/ Vilana, 12  08022 Barcelona, Spain |
| Comite Etico del H. Gral Univ. de Elche  Cami de L'almazara, 11  03203 Elche, Spain |

| Comite Etico de Investigacion Clinica  Hospital Virgen de la Macarena  Avda. Dr. Fedriani, 3  41071 Sevilla, Spain |
| --- |
| Comite Etico del Hospital Arnau de Vilanova  Hospital Arnau de Vilanova  Avda. Alcalde Rovira Roura, 80  25006 Lerida, Spain |
| Comite Etico de Ensayos clinicos  Hospital General Universitario de Alicante  Maestro Alonso, 109  03010 Alicante, Spain |
| Comite Etico de Investigacion Clinica  Hospital de Alarcos (6 planta)  Avda. Pio XII s/n  13002 Ciudad Real, Spain |
| Comite Etico del Hospital Carlos Haya  Avda. Carlos Yaha, s/n  29016 Malaga, Spain |
| Secretaria del Comite etico de Investigacion Clinica  Hospital de Leon  C/ Altos de nava, s/n  24071 Leon, Spain |
| Comite Etico de Investigacion Clinica De Hospital de Jerez de la Frontera  Ctra. De Circunvalacion, s/n  11407 Jerez de la Frontera, Cadiz, Spain |
| Comite Etico del Hospital de Barbastro  Hospital de Barbastro  Ctra nacional 240 Tarragona-San Sebastian  22300 Huesca, Spain |
| Comite Etico de Ensayos clinicos  Hospital General Universitario de Guadalajara  Donantes de Sangre, s/n  19002 Guadalajara, Spain |
| Comite Etico de Investigacion Clinica Hospital General Virgen de la Concha  Avda. Requejo, 35  49003 Zamora, Spain |

| Comite Etico de Investigacion Clinica Complejo Hospitalrio de Toledo Hospital Virgen de la Salud  Avda. De Barber, 30  45004 Toledo, Spain |
| --- |
| Comite Etico del Hospital Dos de Mayo  Hospital Dos de Mayo  C/ Dos de Mayo 301  08025 Barcelona, Spain |
| CEIC  Hospital Universitario Marques de Valdecilla  Avda. De Valdecilla, s/n  39008 Santander, Spain |
| Comite Etico del Hospital Doctor Peset  Valencia, Spain |
| Comite Etico de Investigacion Clinica  Hospital Ciudad de Jaen  Avda. Del Ejercito espanol, s/n  23007 Jaen, Spain |
| Comite etico de Investigacion Clinica  Hospital General San Jorge  Avda. Martinez de Velasco, 36  22004 Huesca, Spain |
| Comite Etico de Investigacion Clinica Hospital Universitario San Cecilio  Avda. Del Dr. Oloriz, 16  18013 Granada, Spain |
| Comite Etico de Ensayos clinicos  Hospital de Bellvitge  Feixa Llarga, s/n  08907 Hospitalet de Llobregat  Barcelona, Spain |
| CEIC Hospital de Txagorritxu  6 planta D-C  01009 Vitoria, Spain |
| Ethics Commission  Kantonsspital, Aarau, Switzerland |

| Ethikkommission bei der Basel  EKBB Hebelstrasse 53  4056 Basel, Switzerland |
| --- |
| Ethik Kommission  St. Gallen, Switzerland |
| Commission de'Ethique de la Sociatel du Valais  70 Rante de Vissigen, 1950 Sion  Switzerland |
| Cantonal Ethics Committee SPUK fur Specialfacher University Hospital of Zurich  8091 Zurich, Switzerland |
| Rue d'Ethique du CHUV Rue du Brignon  Lausanne, Switzerland (CH) |
| Comitato etico cantonale  6501 Bellinzona, Switzerland |

| Commission d'Ethique de la Recherche Clinique  Rue de Bugnon 21  1005 Lausanne, Switzerland |
| --- |
| Ethik Komission Kanton, Zurich Switzerland |
| Comite Departmental d'Ethique De Medecine HUG – Geneve Switzerland |
| Kantonale Ethikkommission Aargau  Kantonsspital Aarau  5001 Aarau, Switzerland |
| Cantonal Ethics Committee  Zurich, Switzerland |
| Ethikkommission des Kantons St. Gallen  Kantonsspital St. Gallen  9007 St. Gallen, Switzerland |
| Kantonale Ethikkommission (KEK)  3010 Bern, Switzerland |
| Kantonale Ethik-Kommission (KEK) SPUK fur Spezialfacher Universitatsspital Zurich Sonneggstrasse 12  8091 Zurich, Switzerland |
| Ethisches Komitee, Universitatsspital Zurich Ramistrasse 100  8091 Zurich  Switzerland |
| Kant. Ethik Kommission Belder, Basel Switzerland |
| KEK  Ethik Komission  Bern, Switzerland |
| Ethical Committee at Kantonsspital Aarau (CH) Switzerland |

| Central Manchester LREC Manchester Health Authority Gateway House  Piccadilly Soth  Manchester M60 7LP, United Kingdom  And  Wrightington, Wigan and Leigh LREC Wigan & Leigh NHS Trust  Royal Albert Edward Infirmary  Blackrock  164 Wigan Lane  Wigan WN1 2LA, United Kingdom |
| --- |
| Salford and Trafford LREC  Salford and Trafford Health Authority  5th Floor, Peel House  Albert Street  Eccles  Manchester M18 0NJ, United Kingdom  And  Wrightington, Wigan and Leigh LREC Wigan & Leigh NHS Trust  Royal Albert Edward Infirmary  Blackrock  164 Wigan Lane  Wigan WN1 2LA, United Kingdom |
| Harrogate Local Research Ethics Committee  Harrogate District Hospital  Strayside Wing  Lancaster Park Road  Harrogate HG2 7SX, United Kingdom |
| Wrightington, Wigan and Leigh LREC Royal Albert Edward Infirmary  Wigan Lane  Wigan WN1 2NN, United Kingdom |

| North West Surrey LREC Bournewood House  St. Peter's Hospital Site  Guildford Road  Chertsey KT16 0QA, United Kingdom |
| --- |
| Northallerton LREC Friarage Hospital Room 59  Trust Headquarters  Northallerton DL6 1JG, United Kingdom  And  Harrogate Health Care NHS Trust LREC Harrogate District Hospital  Strayside Wing  Lancaster Park Road  Harrogate HG2 7SX, United Kingdom |
| South Humber LREC  South Humber Health Authority  Health Place  Wrawby Road  Brigg DN20 8GS, United Kingdom  And  Harrogate Health Care NHS Trust LREC Harrogate District Hospital  Strayside Wing  Lancaster Park Road  Harrogate HG2 7SX, United Kingdom |

| Brighton LREC  Brighton and Hove PCT  6th Floor, Vantage Point  New England Road  Brighton BN1 4GW, United Kingdom  And  North West Surrey LREC Bournewood House  St. Peter's Hospital Site  Guildford Road  Chertsey KT16 0QA, United Kingdom |
| --- |
| Oxford Research Ethics Committee  John Radcliffe Hospital  Research and Development  Room 13, 1st Floor, Manor House  Oxford OX3 9DZ, United Kingdom |
| Hull and East Riding LREC University of Hull  East Riding Campus Coniston House Beverley Road  Willerby HU10 6NS, United Kingdom  And  Harrogate Health Care NHS Trust LREC Harrogate District Hospital  Strayside Wing  Lancaster Park Road  Harrogate HG2 7SX, United Kingdom |
