# Supplementary material for: Safety and effectiveness of adalimumab in patients with rheumatoid arthritis over 5 years of therapy in a phase 3b and subsequent postmarketing observational study
Source: Arthritis Res Ther. 2014 Jan 27;16(1):R24. doi: 10.1186/ar4452 (PMC3979145; doi:10.1186/ar4452)
Supplement: Additional file 3: Table S1-S6 — Overview of serious adverse events and adverse events of interest (E (E/100 PYs)) in patients who recieved no concomitant disease-modifying anti-rheumatic drugs (DMARDs), at least 1 concomitant DMARD, no concomitant corticosteriods, concomitant corticosteriods, no prior infliximab or etanercept, and prior infliximab or etanercept, respectively. E, events; PYs, patient-years. [file ar4452-S3.docx]

**Supplemental table 1** Overview of serious adverse events and adverse events of interest (E[E/100 PYs]) in patients who received no concomitant DMARDs

| **Adverse event (AE)** | **Overall N=1731 (4174 PYs)** | **Time windows after first injection of ADA in ReAct** | | | | |
| --- | --- | --- | --- | --- | --- | --- |
|  |  | **≤0.5 Y**  **N=1731  (775 PYs)** | **>0.5 to 1 Y**  **N=1474**  **(535 PYs)** | **>1 to 3 Y**  **N=1003**  **(1389 PYs)** | **>3 to 5 Y**  **N=586**  **(1005 PYs)** | **>5 Y**  **N=429**  **(469 PYs)** |
| Serious AEs | 727 (17.4) | 294 (37.9) | 135 (25.2) | 165 (11.9) | 101 (10.1) | 32 (6.8) |
| Fatal AEs | 29 (0.7) | 11 (1.4) | 2 (0.4) | 11 (0.8) | 3 (0.3) | 2 (0.4) |
| Serious infections | 128 (3.1) | 46 (5.9) | 23 (4.3) | 34 (2.4) | 22 (2.2) | 3 (0.6) |
| TB* | 5 (0.1) | 3 (0.4) | 1 (0.2) | 1 (0.1) | 0 | 0 |
| Sepsis | 7 (0.2) | 3 (0.4) | 2 (0.4) | 1 (0.1) | 1 (0.1) | 0 |
| Malignancies^†^ | 31 (0.7) | 6 (0.8) | 1 (0.2) | 15 (1.1) | 6 (0.6) | 3 (0.6) |
| Lymphoma | 6 (0.1) | 1 (0.1) | 0 | 2 (0.1) | 2 (0.2) | 1 (0.2) |
| NMSC | 9 (0.2) | 3 (0.4) | 1 (0.2) | 3 (0.2) | 1 (0.1) | 1 (0.2) |
| Serious CHF | 15 (0.4) | 7 (0.9) | 1 (0.2) | 3 (0.2) | 4 (0.4) | 0 |
| Cerebrovascular AEs | 17 (0.4) | 4 (0.5) | 2 (0.4) | 4 (0.3) | 6 (0.6) | 1 (0.2) |
| Serious hepatic events | 20 (0.5) | 6 (0.8) | 5 (0.9) | 6 (0.4) | 3 (0.3) | 0 |

*Including two patients with a positive test for latent TB during ADA therapy.

^†^Excluding lymphoma and NMSC.

ADA, adalimumab; CHF, congestive heart failure; DMARDs, disease-modifying anti-rheumatic drugs; E, events; NMSC, non-melanoma skin cancer; PYs, patient-years; ReAct, the Research in Active Rheumatoid Arthritis phase 3b study; TB, tuberculosis.

**Supplemental table 2** Overview of serious adverse events and adverse events of interest (E[E/100 PYs]) in patients who received at least one concomitant DMARD

| **Adverse event (AE)** | **Overall N=4879  (14099 PYs)** | **Time windows after first injection of ADA in ReAct** | | | | |
| --- | --- | --- | --- | --- | --- | --- |
|  |  | **≤0.5 Y**  **N=4879  \|(2283 PYs)** | **>0.5 to 1 Y**  **N=4448**  **(1721 PYs)** | **>1 to 3 Y**  **N=3280**  **(4760 PYs)** | **>3 to 5 Y**  **N=2037**  **(3544 PYs)** | **>5 Y**  **N=1571**  **(1791 PYs)** |
| Serious AEs | 1802 (12.8) | 544 (23.8) | 284 (16.5) | 496 (10.4) | 316 (8.9) | 162 (9.0) |
| Fatal AEs | 73 (0.5) | 18 (0.8) | 17 (1.0) | 16 (0.3) | 14 (0.4) | 8 (0.4) |
| Serious infections | 390 (2.8) | 116 (5.1) | 60 (3.5) | 120 (2.5) | 59 (1.7) | 35 (2.0) |
| TB* | 30 (0.2) | 8 (0.4) | 10 (0.6) | 7 (0.1) | 4 (0.1) | 1 (0.1) |
| Sepsis | 28 (0.2) | 10 (0.4) | 2 (0.1) | 6 (0.1) | 6 (0.2) | 4 (0.2) |
| Malignancies^†^ | 90 (0.6) | 13 (0.6) | 15 (0.9) | 30 (0.6) | 19 (0.5) | 13 (0.7) |
| Lymphoma | 9 (0.1) | 0 | 0 | 7 (0.1) | 2 (0.1) | 0 |
| NMSC | 34 (0.2) | 5 (0.2) | 1 (0.1) | 14 (0.3) | 10 (0.3) | 4 (0.2) |
| Serious CHF | 32 (0.2) | 8 (0.4) | 5 (0.3) | 9 (0.2) | 9 (0.3) | 1 (0.1) |
| Cerebrovascular AEs | 39 (0.3) | 9 (0.4) | 3 (0.2) | 12 (0.3) | 9 (0.3) | 6 (0.3) |
| Serious hepatic events | 38 (0.3) | 4 (0.2) | 8 (0.5) | 10 (0.2) | 10 (0.3) | 6 (0.3) |

*Including two patients with a positive test for latent TB during ADA therapy.

^†^Excluding lymphoma and NMSC.

ADA, adalimumab; CHF, congestive heart failure; DMARD, disease-modifying anti-rheumatic drug; E, events; NMSC, non-melanoma skin cancer; PYs, patient-years; ReAct, the Research in Active Rheumatoid Arthritis phase 3b study; TB, tuberculosis.

**Supplemental table 3**  Overview of serious adverse events and adverse events of interest (E[E/100 PYs]) in patients who did not receive concomitant corticosteroids

| **Adverse event (AE)** | **Overall N=1902  (5538 PYs)** | **Time windows after first injection of ADA in ReAct** | | | | |
| --- | --- | --- | --- | --- | --- | --- |
|  |  | **≤0.5 Y**  **N=1902  (875 PYs)** | **>0.5 to 1 Y**  **N=1696**  **(650 PYs)** | **>1 to 3 Y**  **N=1243**  **(1882 PYs)** | **>3 to 5 Y**  **N=818**  **(1431 PYs)** | **>5 Y**  **N=632**  **(700 PYs)** |
| Serious AEs | 637 (11.5) | 173 (19.8) | 88 (13.5) | 178 (9.5) | 140 (9.8) | 58 (8.3) |
| Fatal AEs | 29 (0.5) | 10 (1.1) | 3 (0.5) | 8 (0.4) | 8 (0.6) | 0 |
| Serious infections | 129 (2.3) | 42 (4.8) | 16 (2.5) | 34 (1.8) | 26 (1.8) | 11 (1.6) |
| TB* | 10 (0.2) | 4 (0.5) | 4 (0.6) | 1 (0.1) | 1 (0.1) | 0 |
| Sepsis | 8 (0.1) | 1 (0.1) | 1 (0.2) | 1 (0.1) | 4 (0.3) | 1 (0.1) |
| Malignancies^†^ | 29 (0.5) | 5 (0.6) | 5 (0.8) | 13 (0.7) | 3 (0.2) | 3 (0.4) |
| Lymphoma | 2 (0) | 0 | 0 | 1 (0.1) | 1 (0.1) | 0 |
| NMSC | 14 (0.3) | 1 (0.1) | 0 | 7 (0.4) | 4 (0.3) | 2 (0.3) |
| Serious CHF | 19 (0.3) | 4 (0.5) | 1 (0.2) | 5 (0.3) | 9 (0.6) | 0 |
| Cerebrovascular AEs | 15 (0.3) | 3 (0.3) | 1 (0.2) | 5 (0.3) | 4 (0.3) | 2 (0.3) |
| Serious hepatic events | 23 (0.4) | 3 (0.3) | 6 (0.9) | 4 (0.2) | 7 (0.5) | 3 (0.4) |

*Including two patients with a positive test for latent TB during ADA therapy.

^†^Excluding lymphoma and NMSC.

ADA, adalimumab; CHF, congestive heart failure; E, events; NMSC, non-melanoma skin cancer; PYs, patient-years; ReAct, the Research in Active Rheumatoid Arthritis phase 3b study; TB, tuberculosis.

**Supplemental table 4**  Overview of serious adverse events and adverse events of interest (E[E/100 PYs]) in patients who received concomitant corticosteroids

| **Adverse event (AE)** | **Overall N=4708  (12735 PYs)** | **Time windows after first injection of ADA in ReAct** | | | | |
| --- | --- | --- | --- | --- | --- | --- |
|  |  | **≤0.5 Y**  **N=4708  (2184 PYs)** | **>0.5 to 1 Y**  **N=4226**  **(1606 PYs)** | **>1 to 3 Y**  **N=3040**  **(4267 PYs)** | **>3 to 5 Y**  **N=1805**  **(3118 PYs)** | **>5 Y**  **N=1368**  **(1560 PYs)** |
| Serious AEs | 1892 (14.9) | 665 (30.5) | 331 (20.6) | 483 (11.3) | 277 (8.9) | 136 (8.7) |
| Fatal AEs | 73 (0.6) | 19 (0.9) | 16 (1.0) | 19 (0.4) | 9 (0.3) | 10 (0.6) |
| Serious infections | 389 (3.1) | 120 (5.5) | 67 (4.2) | 120 (2.8) | 55 (1.8) | 27 (1.7) |
| TB* | 25 (0.2) | 7 (0.3) | 7 (0.4) | 7 (0.2) | 3 (0.1) | 1 (0.1) |
| Sepsis | 27 (0.2) | 12 (0.5) | 3 (0.2) | 6 (0.1) | 3 (0.1) | 3 (0.2) |
| Malignancies^†^ | 92 (0.7) | 14 (0.6) | 11 (0.7) | 32 (0.7) | 22 (0.7) | 13 (0.8) |
| Lymphoma | 13 (0.1) | 1 (0) | 0 | 8 (0.2) | 3 (0.1) | 1 (0.1) |
| NMSC | 29 (0.2) | 7 (0.3) | 2 (0.1) | 10 (0.2) | 7 (0.2) | 3 (0.2) |
| Serious CHF | 28 (0.2) | 11 (0.5) | 5 (0.3) | 7 (0.2) | 4 (0.1) | 1 (0.1) |
| Cerebrovascular AEs | 41 (0.3) | 10 (0.5) | 4 (0.2) | 11 (0.3) | 11 (0.4) | 5 (0.3) |
| Serious hepatic events | 35 (0.3) | 7 (0.3) | 7 (0.4) | 12 (0.3) | 6 (0.2) | 3 (0.2) |

*Including two patients with a positive test for latent TB during ADA therapy.

^†^Excluding lymphoma and NMSC.

ADA, adalimumab; CHF, congestive heart failure; E, events; NMSC, non-melanoma skin cancer; PYs, patient-years; ReAct, the Research in Active Rheumatoid Arthritis phase 3b study; TB, tuberculosis.

**Supplemental table 5**  Overview of serious adverse events and adverse events of interest (E[E/100 PYs]) in patients who received no prior infliximab or etanercept before first injection of ADA in ReAct

| **Adverse event (AE)** | **Overall N=5711  (16203 PYs)** | **Time windows after first injection of ADA in ReAct** | | | | |
| --- | --- | --- | --- | --- | --- | --- |
|  |  | **≤0.5 Y**  **N=5711  (2657 PYs)** | **>0.5 to 1 Y**  **N=5161**  **(1988 PYs)** | **>1 to 3 Y**  **N=3790**  **(5459 PYs)** | **>3 to 5 Y**  **N=2336**  **(4056 PYs)** | **>5 Y**  **N=1796**  **(2044 PYs)** |
| Serious AEs | 2130 (13.1) | 663(25.0) | 352 (17.7) | 581 (10.6) | 369 (9.1) | 165 (8.1) |
| Fatal AEs | 82 (0.5) | 21 (0.8) | 17 (0.9) | 23 (0.4) | 13 (0.3) | 8 (0.4) |
| Serious infections | 432 (2.7) | 124 (4.7) | 70 (3.5) | 139 (2.5) | 68 (1.7) | 31 (1.5) |
| TB* | 33 (0.2) | 10 (0.4) | 10 (0.5) | 8 (0.1) | 4 (0.1) | 1 (0) |
| Sepsis | 24 (0.1) | 7 (0.3) | 3 (0.2) | 5 (0.1) | 6 (0.1) | 3 (0.1) |
| Malignancies† | 107 (0.7) | 14 (0.5) | 14 (0.7) | 41 (0.8) | 23 (0.6) | 15 (0.7) |
| Lymphoma | 13 (0.1) | 1 (0) | 0 | 8 (0.1) | 3 (0.1) | 1 (0) |
| NMSC | 39 (0.2) | 7 (0.3) | 1 (0.1) | 17 (0.3) | 9 (0.2) | 5 (0.2) |
| Serious CHF | 38 (0.2) | 11 (0.4) | 3 (0.2) | 12 (0.2) | 11(0.3) | 1 (0) |
| Cerebrovascular AEs | 49 (0.3) | 10 (0.4) | 4 (0.2) | 15 (0.3) | 13 (0.3) | 7 (0.3) |
| Serious hepatic events | 51 (0.3) | 7 (0.3) | 13 (0.7) | 13 (0.2) | 13 (0.3) | 5 (0.2) |

*Including two patients with a positive test for latent TB during ADA therapy.

^†^Excluding lymphoma and NMSC.

ADA, adalimumab; CHF, congestive heart failure; E, events; NMSC, non-melanoma skin cancer; PYs, patient-years; ReAct, the Research in Active Rheumatoid Arthritis phase 3b study; TB, tuberculosis.

**Supplemental table 6** Overview of serious adverse events and adverse events of interest (E[E/100 PYs]) in patients who received prior infliximab or etanercept up to 2 months before first injection of ADA in ReAct

| **Adverse event (AE)** | **Overall N=899  (2069 PYs)** | **Time windows after first injection of ADA in ReAct** | | | | |
| --- | --- | --- | --- | --- | --- | --- |
|  |  | **≤0.5 Y**  **N=899  (402 PYs)** | **>0.5 to 1 Y**  **N=761**  **(268 PYs)** | **>1 to 3 Y**  **N=493**  **(690 PYs)** | **>3 to 5 Y**  **N=287**  **(493 PYs)** | **>5 Y**  **N=204**  **(216 PYs)** |
| Serious AEs | 399 (19.3) | 175 (43.6) | 67 (25.0) | 80 (11.6) | 48 (9.7) | 29 (13.4) |
| Fatal AEs | 20 (1.0) | 8 (2.0) | 2 (0.7) | 4 (0.6) | 4 (0.8) | 2 (0.9) |
| Serious infections | 86 (4.2) | 38 (9.5) | 13 (4.8) | 15 (2.2) | 13 (2.6) | 7 (3.2) |
| TB* | 2 (0.1) | 1 (0.2) | 1 (0.4) | 0 | 0 | 0 |
| Sepsis | 11 (0.5) | 6 (1.5) | 1 (0.4) | 2 (0.3) | 1 (0.2) | 1 (0.5) |
| Malignancies^†^ | 14 (0.7) | 5 (1.2) | 2 (0.7) | 4 (0.6) | 2 (0.4) | 1 (0.5) |
| Lymphoma | 2 (0.1) | 0 | 0 | 1 (0.1) | 1 (0.2) | 0 |
| NMSC | 4 (0.2) | 1 (0.2) | 1 (0.4) | 0 | 2 (0.4) | 0 |
| Serious CHF | 9 (0.4) | 4 (0.1) | 3 (1.1) | 0 | 2 (0.4) | 0 |
| Cerebrovascular AEs | 7 (0.3) | 3 (0.7) | 1 (0.4) | 1 (0.1) | 2 (0.4) | 0 |
| Serious hepatic events | 7 (0.3) | 3 (0.7) | 0 | 3 (0.4) | 0 | 1 (0.5) |

*Including two patients with a positive test for latent TB during ADA therapy.

^†^Excluding lymphoma and NMSC.

ADA, adalimumab; CHF, congestive heart failure; E, events; NMSC, non-melanoma skin cancer; PYs, patient-years; ReAct, the Research in Active Rheumatoid Arthritis phase 3b study; TB, tuberculosis.
