# Supplementary material for: Safety and effectiveness of adalimumab in patients with rheumatoid arthritis over 5 years of therapy in a phase 3b and subsequent postmarketing observational study
Source: Arthritis Res Ther. 2014 Jan 27;16(1):R24. doi: 10.1186/ar4452 (PMC3979145; doi:10.1186/ar4452)
Supplement: Additional file 4: Figure S1 — Percentages of patients with American College of Rheumatology (ACR) (A) 20%, (B) 50%, or (C) 70% improvement for those patients with prior use of TNF antagonists and TNF antagonist-naive patients. Data are shown as observed values for all evaluable patients at each time point during long-term treatment with adalimumab (ADA). LO, last observation. [file ar4452-S4.pdf]

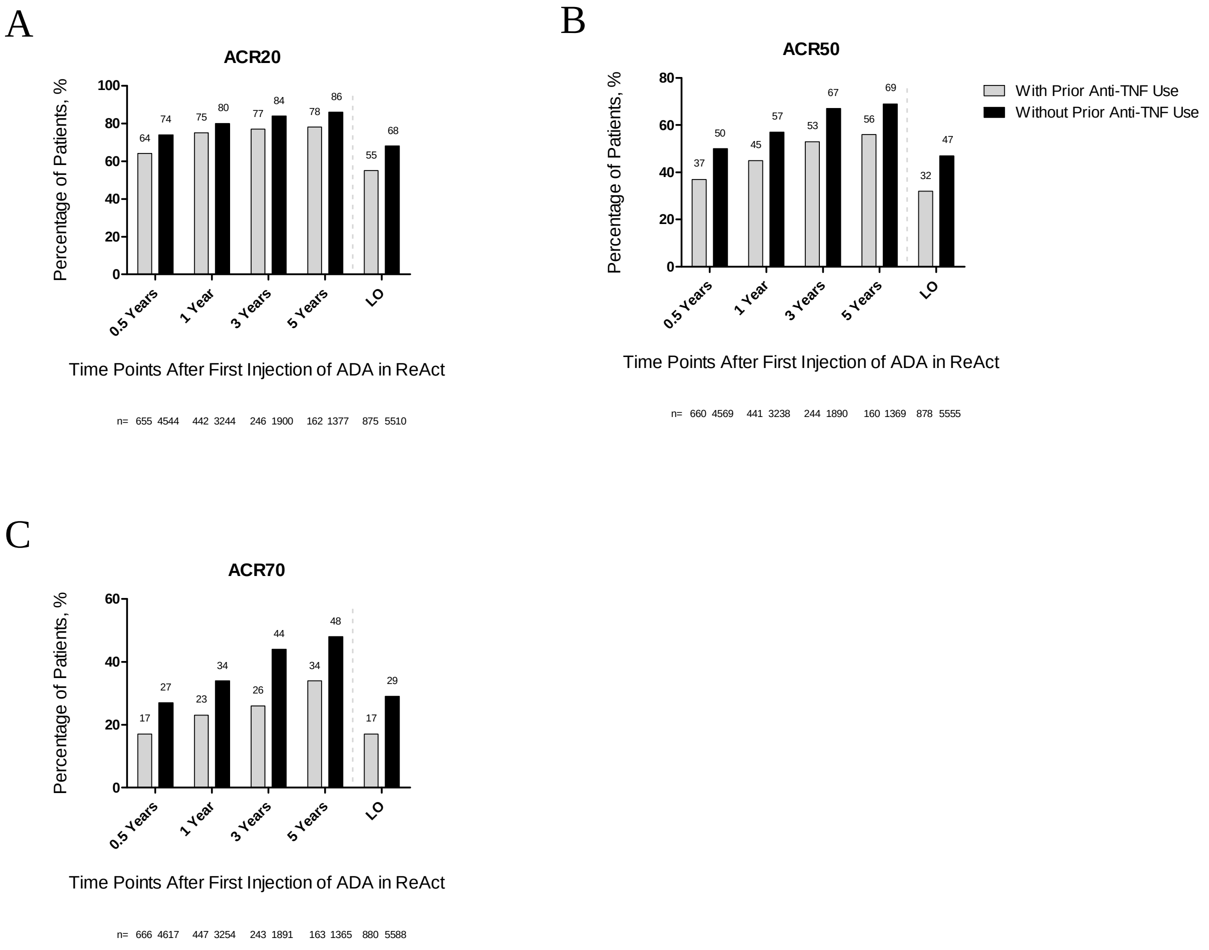

Supplemental Figure 1 Percentages of patients with American College of Rheumatology (ACR) (A) 20%, (B) 50%, or (C) 70% improvement for those patients with prior use of TNF antagonists and TNF naïve patients. Data are shown as observed values for all evaluable patients at each time point during long-term treatment with adalimumab (ADA). LO, last observation.
