# Supplementary material for: Safety and effectiveness of adalimumab in patients with rheumatoid arthritis over 5 years of therapy in a phase 3b and subsequent postmarketing observational study
Source: Arthritis Res Ther. 2014 Jan 27;16(1):R24. doi: 10.1186/ar4452 (PMC3979145; doi:10.1186/ar4452)
Supplement: Additional file 5: Figure S2 — European League Against Rheumatism (EULAR) responses of at least ‘moderate’ and ‘good’ for those patients with prior use of TNF antagonists and TNF antagonist-naive patients. Data are shown as observed values for all evaluable patients at each time point during long-term treatment with adalimumab (ADA). LO, last observation. [file ar4452-S5.pdf]

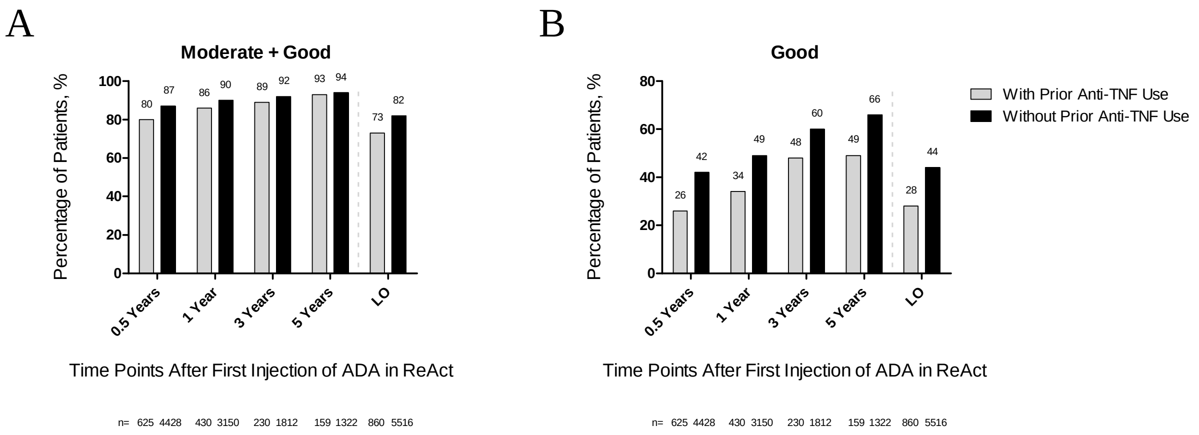

Supplemental Figure 2 European League Against Rheumatism (EULAR) responses of at least ‘moderate’ and ‘good’ for those patients with prior use of TNF antagonists and TNF naïve patients. Data are shown as observed values for all evaluable patients at each time point during long-term treatment with adalimumab (ADA). LO, last observation.
