# Supplementary material for: Safety and effectiveness of adalimumab in patients with rheumatoid arthritis over 5 years of therapy in a phase 3b and subsequent postmarketing observational study
Source: Arthritis Res Ther. 2014 Jan 27;16(1):R24. doi: 10.1186/ar4452 (PMC3979145; doi:10.1186/ar4452)
Supplement: Additional file 6: Figure S3 — (A) Low disease activity (LDA) and (B) disease remission (REM) defined as Simplified Disease Activity Index (SDAI) ≤11 and SDAI ≤3.3, respectively, for those patients with prior use of TNF antagonists and TNF antagonist-naive patients. Percentages of patients with (C) minimal important difference ≥0.22 on the Health Assessment Questionnaire Disability Index (HAQ DI) and (D) normal function defined as HAQ DI ≤0.5. Data are shown as observed values for all evaluable patients at each time point during long-term treatment with adalimumab (ADA). LO, last observation. [file ar4452-S6.pdf]

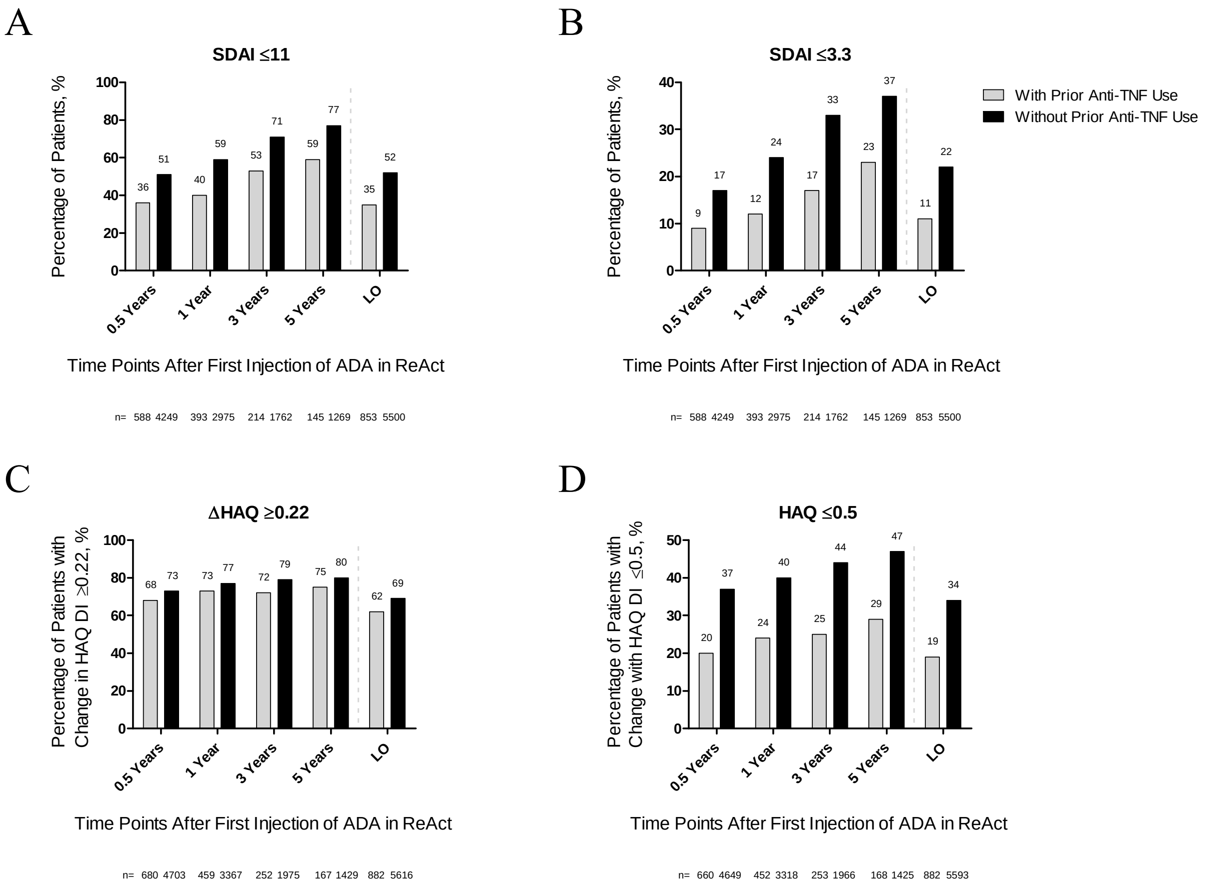

Supplemental Figure 3 (A) Low disease activity (LDA) and (B) disease remission (REM) defined as Simplified Disease Activity Index (SDAI) ≤11 and SDAI ≤3.3, respectively, for those patients with prior use of TNF antagonists and TNF naïve patients. Percentages of patients with (C) minimal important difference ≥0.22 on the Health Assessment Questionnaire Disability Index (HAQ DI) and (D) normal function defined as HAQ DI ≤0.5. Data are shown as observed values for all evaluable patients at each time point during long-term treatment with adalimumab (ADA). LO, last observation.
